# Catch-and-Release: The Assembly, Immobilization, and Recycling of Redox-Reversible Artificial Metalloenzymes

**DOI:** 10.1021/acscatal.3c05294

**Published:** 2024-02-15

**Authors:** Alex H. Miller, Elena V. Blagova, Benjamin Large, Rosalind L. Booth, Keith S. Wilson, Anne-K. Duhme-Klair

**Affiliations:** † Department of Chemistry, University of York, Heslington, York YO10 5DD, U.K.; ‡ Structural Biology Laboratory, Department of Chemistry, University of York, Heslington, York YO10 5DD, U.K.

**Keywords:** artificial metalloenzymes, immobilization, recyclability, reversible cofactor anchoring, siderophores

## Abstract

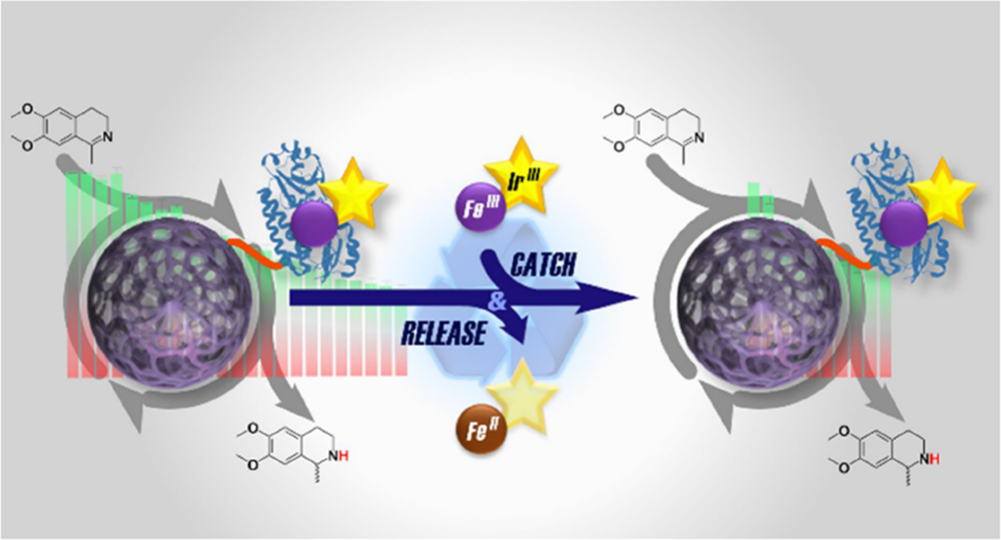

Technologies to improve the applicability of artificial metalloenzymes (ArMs) are gaining considerable interest; one such approach is the immobilization of these biohybrid catalysts on support materials to enhance stability and enable their retention, recovery, and reuse. Here, we describe the immobilization of polyhistidine-tagged ArMs that allow the redox-controlled replacement of catalytic cofactors that have lost activity, e.g., due to poisoning or decomposition, on immobilized metal affinity chromatography resins. By using periplasmic siderophore-binding protein scaffolds that originate from thermophilic bacteria (***Gst***CeuE and ***Pth***CeuE) in combination with a siderophore-linked imine reduction catalyst, reaction rates were achieved that are about 3.5 times faster than those previously obtained with ***Cj***CeuE, the analogous protein of *Campylobacter jejuni*. Upon immobilization, the ***Gst***CeuE-derived ArM showed a decrease in turnover frequency in the reduction of dehydrosalsolidine by 3.4-fold, while retaining enantioselectivity (36%) and showing improved stability that allowed repeat recovery and recycling cycles. Catalytic activity was preserved over the initial four cycles. In subsequent cycles, a gradual reduction of activity was evident. Once the initial activity decreased to around 40% of the initial activity (23rd recycling cycle), the redox-triggered artificial cofactor release permitted the subsequent recharging of the immobilized protein scaffold with fresh, active cofactor, thereby restoring the initial catalytic activity of the immobilized ArM and allowing its reuse for several more cycles. Furthermore, the ArM could be assembled directly from protein present in crude cell extracts, avoiding time-consuming and costly protein purification steps. Overall, this study demonstrates that the immobilization of redox-reversible ArMs facilitates their “catch-and-release” assembly and disassembly and the recycling of their components, improving their potential commercial viability and environmental footprint.

## Introduction

Artificial metalloenzymes (ArMs) are hybrid systems that combine the catalytic properties of synthetic metal complexes with the structural and functional properties of proteins. These hybrid systems serve the purpose of facilitating the utilization of efficient synthetic catalysts under biocompatible conditions, which frequently encounters challenges, such as catalyst instability in aqueous biological media. Moreover, ArMs offer the added advantage of improved (enantio)selectivity. Leveraging the inherent features of genetically evolvable protein scaffolds, ArMs strive to enable new-to-nature biocompatible reactions.^1−6^

Despite significant advances in the field, the applications of ArMs are mostly limited to laboratory-scale reactions, in part due to the high costs involved in the scaffold and cofactor preparation, combined with low stability, low catalytic efficiency, and challenging scalability.^7,8^ Similar challenges in the applications of natural enzymes are frequently addressed by taking advantage of immobilization techniques.^9^ Immobilization does not only facilitate product isolation and subsequent enzyme recycling, but it can also improve both the thermal stability and organic solvent tolerance of enzymes,^10,11^ considerations that are key to overcoming the high costs and barriers associated with the application of enzymes in many biocatalytic processes.^9,12−27^

**Figure 1:**
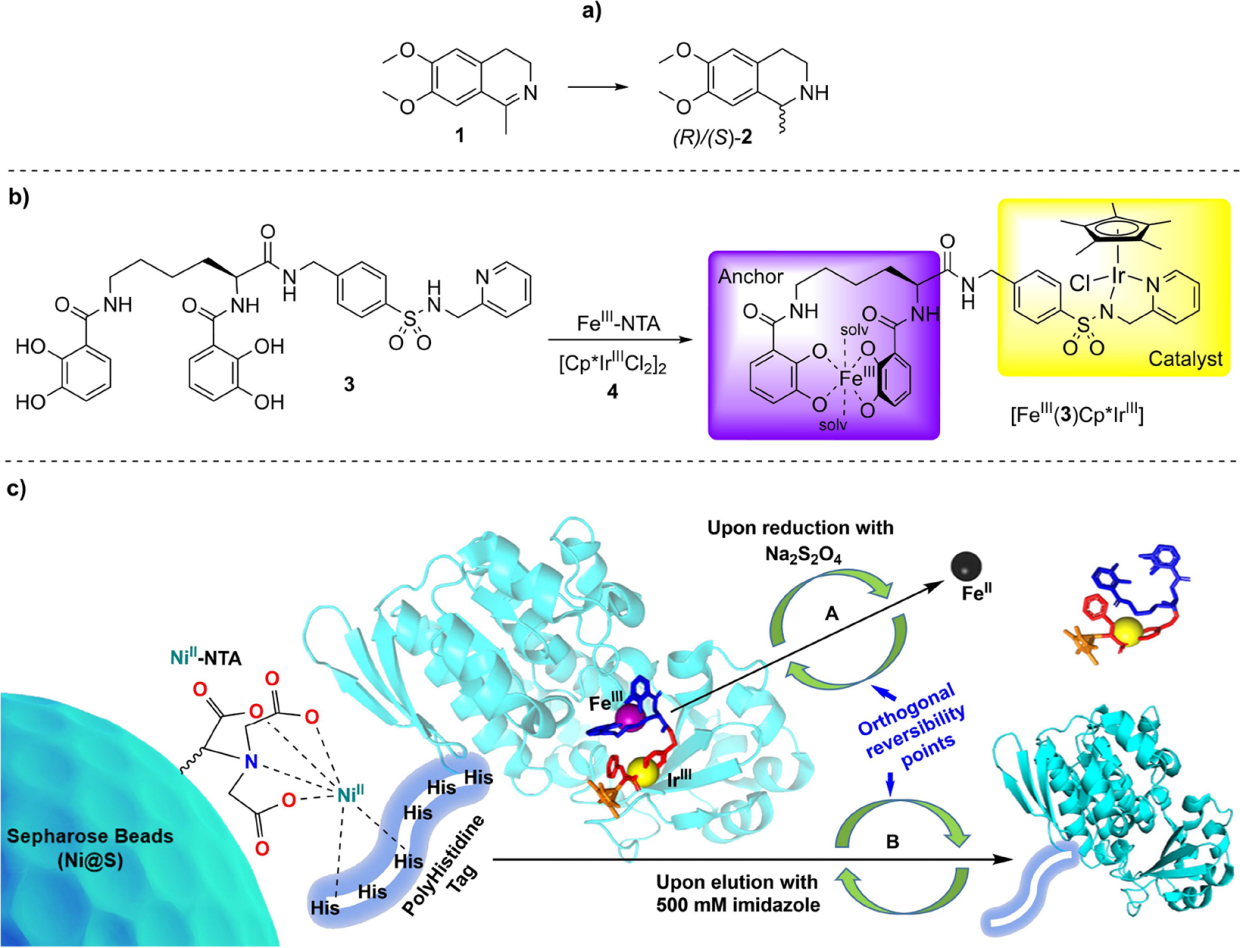
Asymmetric transfer hydrogenation reaction, ArM (PDB: 5OD5) and immobilization strategy used in this study. (a) Transfer hydrogenation of pro-chiral imine (**1**) to (*R*)/(*S*)-salsolidine (**2**). (b) Reaction of siderophore-catalyst conjugate (**3**) with iron-nitrilotriacetic acid (Fe^III^–NTA) and [Cp*Ir^III^Cl_2_]_2_ (**4**) to form the heterodinuclear complex [Fe^III^(solv)_2_(**3**)Cp*Ir^III^Cl]^−^, termed [Fe^III^(**3**)Cp*Ir^III^] in the text. (c) From left to right: Polyhistidine-tagged ArM immobilized on Ni-NTA-modified Sepharose beads (Ni@S). (A) Cofactor release via reduction of Fe^III^ to Fe^II^ with sodium dithionite (Na_2_S_2_O_4_). (B) Cleavage of the polyhistidine-tagged protein from Ni@S by the use of an imidazole gradient.

Recently, the advantages of enzyme immobilization have motivated similar approaches in the field of ArMs.^28−38^ Tagging strategies, for example, allowed natural protein–ligand affinities to be exploited for the irreversible^39^ or reversible^40,41^ immobilization on microbeads. The scope of reactions to which immobilized ArMs have been applied is broad, ranging from Diels–Alder,^34^ C–C cross-coupling,^33^ and cyclopropanation reactions^41^ to oxidative^30−32,36,37,39^ and reductive transformations.^28,35,38^ Of these, the reduction of pro-chiral imines via transfer hydrogenation has attracted substantial interest (Figure 1a), since it is widely used in the pharmaceutical industry to produce high-value chiral amines.^4,42,43^

In 2015 Hestericová et al.^28^ reported the covalent immobilization of a streptavidin-derived artificial transfer hydrogenase (ATHase) on silica nanoparticles and achieved remarkable activity and enantioselectivity in the production of (*R*)/(*S*)*-*salsolidine (**2**). The immobilized ATHase showed good stability in crude cellular extracts and moderate recyclability. In 2018, a different approach was reported by Hestericová et al.^38^ in which a streptavidin-derived ATHase was entrapped in ferritin cages, leading to improved turnover numbers associated with the accumulated effects of both secondary and tertiary coordination spheres from streptavidin and ferritin, respectively.

While the immobilization of ArMs in these systems led to improvements in stability and overall applicability, in most cases in which recyclability was investigated, a gradual reduction in activity was observed over repeated cycles, mainly due to cofactor inactivation. A common feature among these ArMs is the irreversible anchoring of the metal cofactors to their protein scaffold, which ties the artificial enzyme lifetime to the stability of its cofactor, even if the protein scaffold remains intact after several runs. Ideally, a reversible anchoring strategy is needed to permit the recharging of the scaffold with an active cofactor as required, and this is the approach investigated in this study. We previously developed an artificial transfer hydrogenase (ATHase) that consists of a Fe^III^-siderophore-anchored iridium-based transfer hydrogenation catalyst, hereafter referred to as [Fe^III^(**3**)Cp*Ir^III^] (Figure 1b), bound to CeuE, a periplasmic-binding protein (PBP) from *Campylobacter jejuni* (***Cj***CeuE).^2^ Our approach exploits the high affinity that siderophores, Fe^III^-chelating molecules that mediate microbial iron uptake, have for their cognate PBPs. The unique feature of the Fe^III^-siderophore-based anchor is that it enables the iridium-based catalyst to be bound strongly, yet reversibly, to the protein scaffold. After deactivation of the iridium-based catalyst, the ATHase can be disassembled by reduction of Fe^III^ to Fe^II^, which triggers the dissociation of the components and permits the recovery and reuse of the protein scaffold.

Herein, we describe the immobilization of our redox-reversible ATHases via their protein scaffold in a way that provides two orthogonal points of reversibility (Figure 1c), enabling the “catch-and-release” of either the catalytic cofactor or the protein, as required, and allowing the recovery and reuse of the individual components.

## Results and Discussion

### Design Considerations

We were interested in an immobilization platform that would allow us to expand the catch-and-release capabilities of our system. Our aim was to find a solution that provided an orthogonal point of reversibility, allowing the modification and manipulation of the immobilized entities as needed. Additionally, we sought a platform that incorporated commercially available carrier materials, ensuring that the approach could be readily implemented.

An interesting strategy was reported by Kato et al.,^40^ who developed a high-throughput screening platform using a maltose-binding protein (mbp) tag to immobilize an ArM consisting of nitrobindin as the protein scaffold and a Cp*Rh^III^-based C–H activation catalyst. The mbp tag (fused with nitrobindin) exploits the affinity of mbp for maltose, present in starch-containing agarose-microbeads, and it can be reversibly detached using an excess of maltose. The mbp-nitrobindin-Cp*Rh^III^ ArM was then utilized after elution; catalytic tests with the ArM while immobilized were not reported.

Our choice of affinity tag was inspired by immobilized metal affinity chromatography (IMAC),^44^ which is routinely used for protein purification. The affinity of polyhistidine tags for metal cations, in particular, Ni^II^, is exploited. In this case, the binding interactions can be reverted using high concentrations of imidazole, which competes with the histidine residues in the tag for Ni^II^ binding, leading to the elution of the tagged protein.

IMAC resins have shown promise as supports for natural enzymes in heterogeneous biocatalytic processes,^15,21,23,24,26^ on occasions capturing the tagged-enzymes straight from cell lysates and avoiding time-consuming and expensive purification steps,^16,22,25,41^ but often with the disadvantage of erosion in activity due to enzyme leakage between reaction cycles. Interestingly, Hao et al.^41^ utilized Ni^II^-nitrilotriacetic acid (Ni-NTA)-based IMAC resin in 96-well plates to create an on-bead screening platform that benefitted greatly from the immobilization of artificial cyclopropanase scaffolds that had been generated via random mutagenesis.

Inspired by the applicability of commercially available Ni^II^–NTA-functionalized solid supports (Ni@S) to on-bead catalysis and the desirable feature of immobilization directly from cell lysates, an *N*-terminal polyhistidine tagging strategy was chosen for the immobilization of our ATHases, Figure 1c. In the crystal structure of ***Cj***CeuE, the *N*-terminal residues are positioned well away from the siderophore-binding cleft, allowing the ArM to be immobilized in the correct “open” orientation. When desired, the immobilized ArM can be liberated from the Ni@S support using an imidazole gradient, enabling the solid supports to be regenerated. Alternatively, the Fe^III^-siderophore anchored catalyst can be released by reduction to Fe^II^, leaving the ***Cj***CeuE scaffold attached to the Ni@S support and ready to be recharged with fresh catalyst. Since the NTA-bound Ni^II^ on the resin is more difficult to reduce than the siderophore-bound Fe^III^, the Ni@S immobilization technique provides a second, orthogonal point of reversibility.

### CeuE Homologs from Thermophilic Organisms as ATHase Scaffolds

In addition to using ***Cj***CeuE as the protein scaffold in ATHases, we employed two recently cloned and overexpressed thermostable homologs, ***Gst***CeuE and ***Pth***CeuE, from the thermophilic organisms *Geobacillus stearothermophilus* and *Parageobacillus thermoglucosidasius*, respectively.^45^ The dissociation constants (*K*_d_) of [Fe^III^(**3**)Cp*Ir^III^] ⊂ ***Gst***CeuE and [Fe^III^(**3**)Cp*Ir^III^] ⊂ ***Pth***CeuE, as measured by intrinsic fluorescence quenching (Table 1, Figure S1), confirmed strong binding; both were lower (by about 2-fold for *Gst*) than the dissociation constant obtained for [Fe^III^(**3**)Cp*Ir^III^] ⊂ ***Cj***CeuE. Subsequently, both ATHases were tested for the reduction of **1** under conditions analogous to those previously reported for ***Cj***CeuE (Table 1, entry 1).^2^ Interestingly, both [Fe^III^(**3**)Cp*Ir^III^] ⊂ ***Gst***CeuE and [Fe^III^(**3**)Cp*Ir^III^] ⊂ ***Pth***CeuE catalyzed the transfer hydrogenation reaction ∼3.5-fold faster than [Fe^III^(**3**)Cp*Ir^III^]⊂***Cj***CeuE, while a moderate reduction in enantiomeric excess (e.e.) was observed (Table 1, entries 2 and 3). Motivated by their improved binding affinities and faster reaction rates, [Fe^III^(**3**)Cp*Ir^III^] ⊂ ***Gst***CeuE and [Fe^III^(**3**)Cp*Ir^III^] ⊂ ***Pth***CeuE were included in our subsequent immobilization studies, in the hope that these ArMs would exhibit a higher catalytic activity than the ***Cj***CeuE-based ATHase and offer enhanced thermostability and organic solvent tolerance.

**Table 1:**
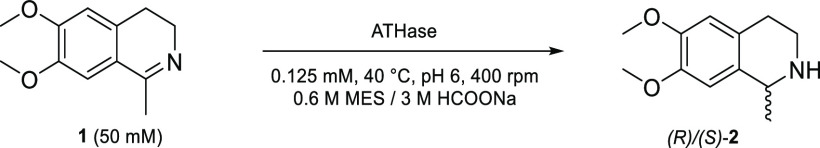
Dissociation Constants (*K*_d_) of ***Cj***CeuE, ***Gst***CeuE and ***Pth***CeuE to [Fe^III^(**3**)Cp*Ir^III^] and Conversion of **1** to (*R*)/(*S*)-**2** Using the Respective ATHases in Homogeneous Catalysis

entry	scaffold	*K*_d_ (nM)	ATHase	time to completion (h)	TON/TOF (min^–1^)	(*R*)-**2** e.e. %
1	***Cj***CeuE	23.4 ± 3.2	[Fe^III^(**3**)Cp*Ir^III^] ⊂ ***Cj***CeuE	24	400/0.30^a^	35
2	***Gst***CeuE	9.9 ± 0.9	[Fe^III^(**3**)Cp*Ir^III^] ⊂ ***Gst***CeuE	7	400/1.71^b^	29
3	***Pth***CeuE	18 ± 1.7	[Fe^III^(**3**)Cp*Ir^III^] ⊂ ***Pth***CeuE	7	400/1.71^b^	24

a TOF calculated at 8 h.^2^

b TOF calculated at 3 h.

### Immobilization and Batch Reaction Setup

**Figure 2:**
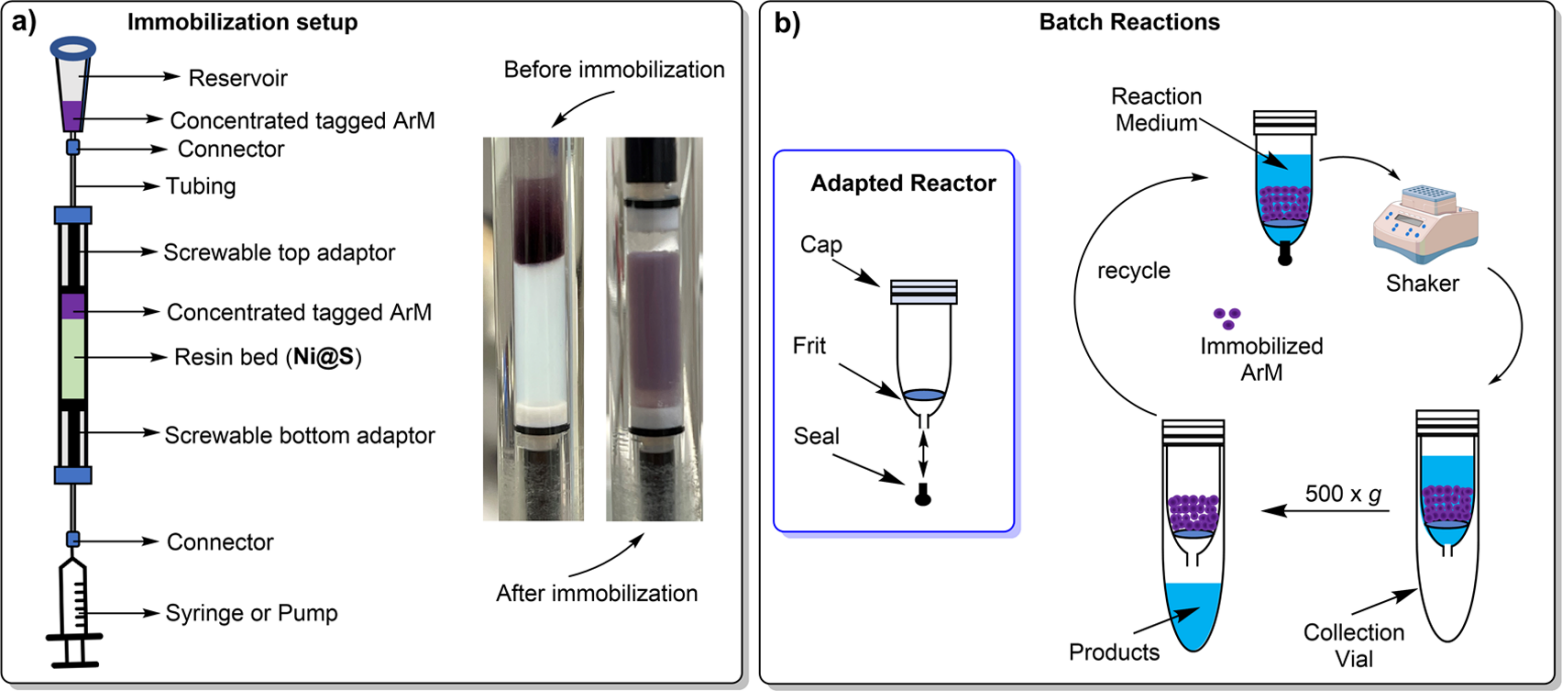
Setup for tagged ArM immobilization and batch reactions. (a) Mono Q column customized for immobilizing ArMs. The column can be connected to either a syringe or a peristaltic pump. The size of the resin bed is adjustable and can be compressed using screwable adaptors at the top and bottom. The immobilized ArM can be unpacked and utilized in batch reactions. (b) Reaction vial modified from commercially available His Spin Trap columns. The sealed and capped reactor is compatible with an Eppendorf shaker, allowing control over the temperature and shaking. Frits are employed to separate ArM beads from the reaction medium through centrifugation. The ArM-Ni@S complex can be easily recycled.

Aiming at a versatile experimental setup for the immobilization of 6His-tagged ATHases, an ion-exchange column (Mono-Q type) was adapted and packed with Ni@S resin for the immobilization of the ArMs, Figure 2a, and detailed in the experimental section. Upon loading with the respective ArM (400 nmol ArM per 1 mL of resin slurry), the color of the IMAC resin changes from light green to purple due to the characteristic color of the iron-siderophore anchor, an integral component of the ArM (Figure 2a). For characterization, the loaded resin was removed from the column and its iron and iridium content analyzed by ICP-OES (Table S1, entries 1–3). The Fe/Ir ratio for [Fe^III^(**3**)Cp*Ir^III^] was found to be close to 1, and this ratio was maintained after ArM assembly to give [Fe^III^(**3**)Cp*Ir^III^] ⊂ 6His-***Gst***CeuE, as well as after immobilization of the ArM to produce [Fe^III^(**3**)Cp*Ir^III^] ⊂ 6His-***Gst***CeuE-Ni@S.

The ArM-loaded resin can be used to catalyze reactions either in flow (inside the column) or in batch reactions (after unpacking). To enable the comparison with nonimmobilized ArMs, we opted to unpack the ArM-Ni@S and carry out investigations in batch, since this allows the catalytic reactions to be performed in parallel using very similar conditions. To facilitate the separation of ArM-Ni@S from the reaction medium, His SpinTrap columns were adapted as reaction vials, Figure 2b. These small columns fit into conventional Eppendorf shakers in which the temperature and shaking conditions can be varied. At the end of each reaction cycle, the reactor (with bottom sealer removed) is placed in an Eppendorf collection vial and centrifuged at low speed. The microporous frit separates the ArM-Ni@S resin from the reaction medium, and new reaction cycles can be started immediately. Additionally, this reaction setup can be used for the redox-triggered disassembly of the ArM, as described in the supplementary methods.

### Batch Reaction Optimization

**Figure 3:**
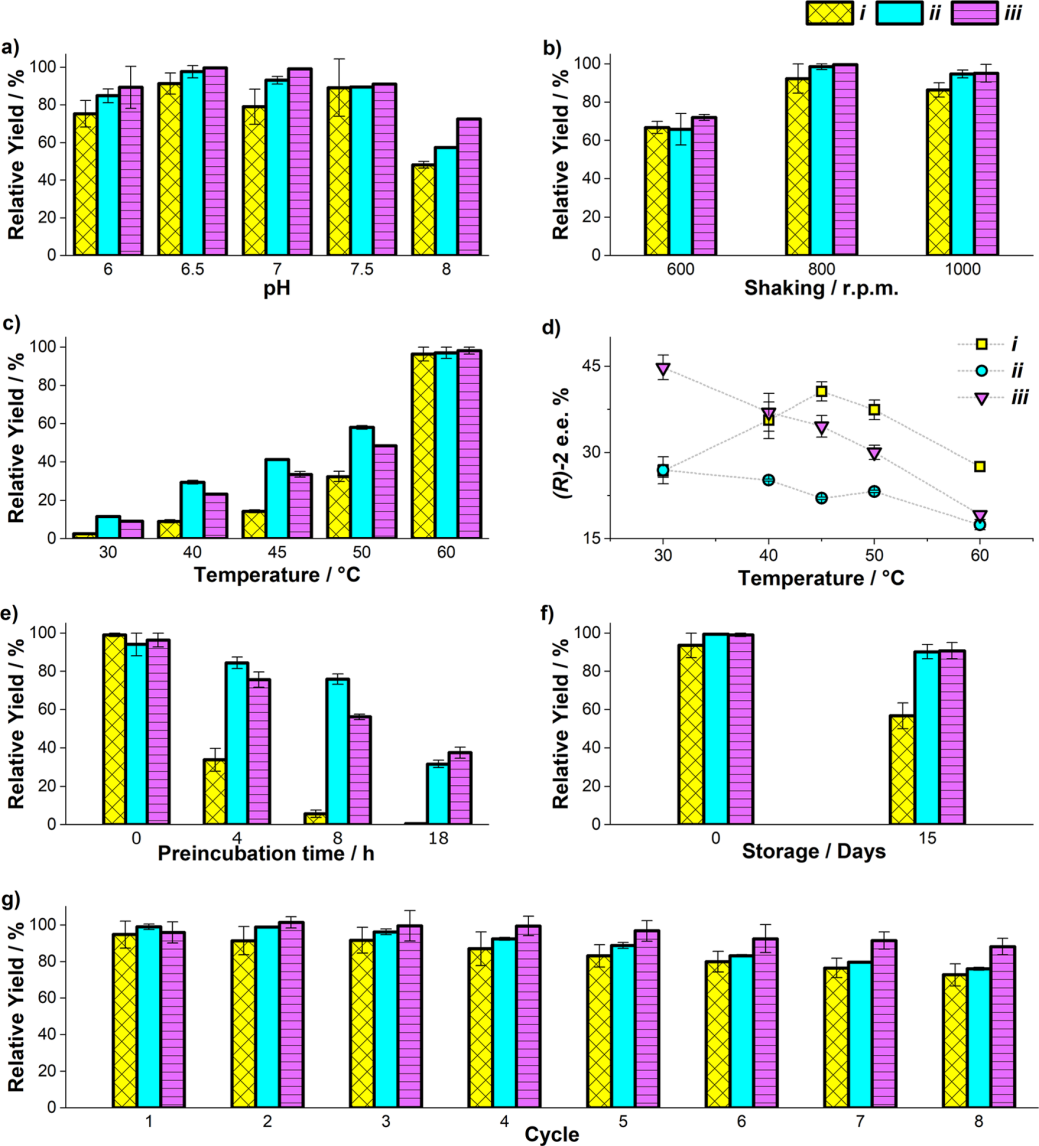
Optimization of batch reaction conditions using immobilized ArMs. Catalytic performance of [Fe^III^(**3**)Cp*Ir^III^] ⊂ 6His-***Cj***CeuE-Ni@S (i), [Fe^III^(**3**)Cp*Ir^III^] ⊂ 6His-***Pth***CeuE-Ni@S (ii) and [Fe^III^(**3**)Cp*Ir^III^] ⊂ 6His-***Gst***CeuE-Ni@S (iii) for the reduction of **1** to (*R*)/(*S*)-**2** achieved (a) between pH 6 and 8, 50 °C, 800 rpm, (b) shaking speeds between 600 and 1000 rpm, 50 °C, pH 7, (c) temperatures between 30 and 60 °C, pH 7, 800 rpm. (d) Enantiomeric excess in favor of the (*R*)-**2** enantiomer obtained at temperatures between 30 and 60 °C. (e) Catalytic performance at 60 °C, pH 7, 800 rpm after ATHase preincubation at 60 °C for 0, 4, 8, and 18 h. (f) Storage stability at <8 °C, pH 7, 50 °C, 800 rpm. (g) Recyclability at 45 °C, pH 7, and 800 rpm. Substrate concentration: 2 mM. Catalyst: 25 μM (1.25 mol %). Catalytic buffer: 0.6 M MES/3 M HCOONa/(desired pH adjusted with NaOH). Error bars show the mean absolute deviation.

A systematic investigation of the three 6His-tagged ATHases ([Fe^III^(**3**)Cp*Ir^III^] ⊂ 6His-***Cj***CeuE-Ni@S, [Fe^III^(**3**)Cp*Ir^III^] ⊂ 6His-***Pth***CeuE-Ni@S and [Fe^III^(**3**)Cp*Ir^III^] ⊂ 6His-***Gst***CeuE-Ni@S) immobilized on Ni@S resin was carried out to establish the influence of pH, shaking speed, temperature, and preincubation conditions on the conversion of **1** to (*R*)/(*S*)-**2** and the enantiomeric excess (Figure 3a–e). To be able to compare the performance of the three immobilized ATHases directly, the total catalyst concentrations were kept constant throughout the experiment. Therefore, centrifugation and washing steps were not performed after the preincubation, prior to starting the activity testing.

A pH range of 6–8 was chosen to investigate the effect of the protonation state of the histidine residues (p*K*_a_ ∼ 6) that fulfill key functions in both the immobilization and catalytic performance of the ATHases.^46^ The protonation of the histidine residues in the His-tag would result in the cleavage of the ArM from the resin, while the protonation of the Ir-coordinating His277 residue in ***Cj***CeuE would compromise the binding of the catalytic cofactor.^2^ In contrast, the nitrogen of the substrate should remain protonated (p*K*_a_ ∼ 7), thereby allowing the buffered aqueous solution to act as the proton source during the transfer hydrogenation reaction.^2^ For the three ArMs tested, optimum activities were between pH 6.5 and pH 7.0 (Figure 3a) with a shaking speed of 800 rpm at pH 7.0 (Figure 3b).

As anticipated, the substrate conversion increases with increasing temperatures (Figure 3c). For both [Fe^III^(**3**)Cp*Ir^III^] ⊂ 6His-***Pth***CeuE-Ni@S and [Fe^III^(**3**)Cp*Ir^III^] ⊂ 6His-***Gst***CeuE-Ni@S, the e.e. decreased with increasing temperatures, as expected (Figure 3d), while for the [Fe^III^(**3**)Cp*Ir^III^] ⊂ 6His-***Cj***CeuE-Ni@S, the e.e. increased up to a temperature of 45 °C but decreased upon further heating (Figure 3d). Similar behavior was reported by Skander et al.^47^ in which the e.e. peaked at 30 °C. Variation in heat-induced entropic effects in ***Cj***CeuE-ATHase compared to ***Gst***CeuE and ***Pth***CeuE aligns well with structural differences reported previously,^45^ in particular the fact that the flexibility of the loop with the Ir-coordinating His227 in ***Cj***CeuE is reduced in the thermophilic scaffolds by H-bonding to a tyrosine residue. In ***Cj***CeuE, the equivalent position is occupied by a nonhydrogen-bonding phenylalanine residue.

Thermostability assays confirmed that ***Gst***CeuE and ***Pth***CeuE provide more stable immobilized ATHases than ***Cj***CeuE. After 18 h of preincubation at 60 °C, both retain over 30% of their catalytic activities and preserve their enantioselectivity, while ***Cj****CeuE* shows an almost complete loss of activity (Figures 3e and S2). This behavior correlates with the thermostability of ***Gst***CeuE and ***Pth***CeuE: their thermal unfolding transition midpoints are about 20 °C higher than that of ***Cj****CeuE*.^45^ The observed decrease in catalytic activity therefore points toward heat-induced ATHase degradation. The leaking of intact catalytically active cofactor from the protein scaffold can be excluded since it would have increased the product yield and reduced the enantioselectivity. The enantiomeric excess, however, was preserved (Figure S2). For [Fe^III^(**3**)Cp*Ir^III^] ⊂ 6His-***Gst***CeuE-Ni@S, the enantioselectivity even increased significantly.

In a control experiment, the free cofactor [Fe^III^(**3**)Cp*Ir^III^] was incubated in solution for 18 h in the absence of protein (Figure S3), and a reduction in catalytic activity by ∼20% was observed. The deactivation of the free catalyst cofactor (∼20%), however, was far less pronounced than that observed with the immobilized ATHases (70% or more). Taken together, these observations point toward the presence of some dissociated or nonspecifically bound catalyst that degrades upon prolonged incubation at high temperature, thereby contributing to the decrease in catalytic activity of the immobilized system. Nevertheless, the deactivation of assembled ATHases appears to be the major factor. The fact that the two thermostable analogs withstand high temperatures for longer than ***Cj***CeuE points toward the involvement of the protein scaffold in the deactivation/denaturation process. When stored at <8 °C for 15 days, both thermophilic analogs retain above 90% of their activities (Figure 3f).

Improved thermostability and increased pH tolerance after immobilization were also observed when comparing the performance of [Fe^III^(**3**)Cp*Ir^III^] ⊂ 6His-***Gst***CeuE-Ni@S with the nonimmobilized ATHase [Fe^III^(**3**)Cp*Ir^III^] ⊂ ***Gst***CeuE (Figure S4). The activity of the nonimmobilized ATHase peaks at pH 6.5 and falls to 80% at pH 6 or pH 7 (Figure S4a). In contrast, after immobilization, 90 and 100% of the activity of the immobilized ATHase is retained at pH 6 and pH 7, respectively (Figure 3a). Furthermore, a complete erosion in enantioselectivity is observed at 60 °C for [Fe^III^(**3**)Cp*Ir^III^] ⊂ ***Gst***CeuE (Figure S4b) in solution, while the immobilized ATHase achieves around 20% (*R*)-**2** e.e. at the same temperature (Figure 3d). Improvements in solvent tolerance and thermal stability are phenomena commonly observed with natural enzymes,^10,11,24,26^ and the trend has also been reported for ArMs after immobilization.^34,36^

Additionally, [Fe^III^(**3**)Cp*Ir^III^] ⊂ 6His-***Cj***CeuE-Ni@S, [Fe^III^(**3**)Cp*Ir^III^] ⊂ 6His-***Pth***CeuE-Ni@S and [Fe^III^(**3**)Cp*Ir^III^] ⊂ 6His-***Gst***CeuE-Ni@S could be recycled and retained 71, 76, and 90% of their initial activities, respectively, after eight consecutive runs (Figure 3g). For the subsequent reversibility studies, the most stable immobilized ArM overall, [Fe^III^(**3**)Cp*Ir^III^] ⊂ 6His-***Gst***CeuE-Ni@S, was chosen.

### Recyclability, Catalyst Release, and Immobilized ArM Reassembly

**Figure 4:**
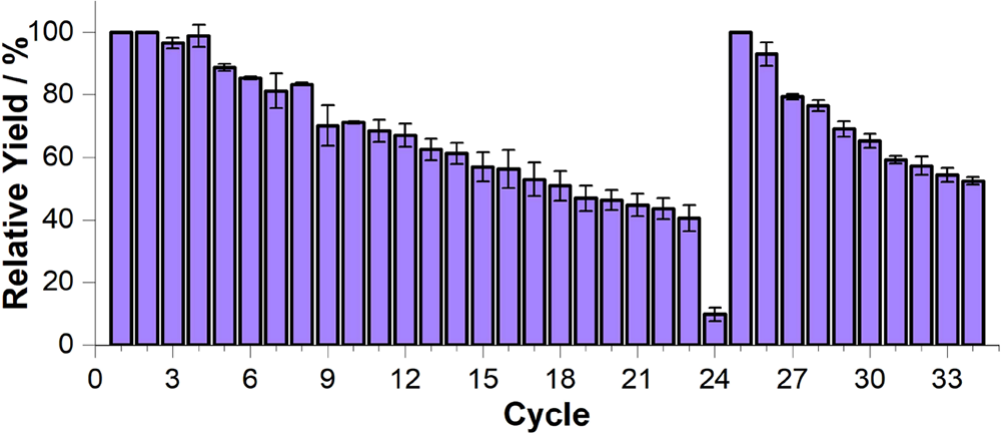
Recyclability and redox-triggered ArM disassembly/reassembly of [Fe^III^(**3**)Cp*Ir^III^] ⊂ 6His-***Gst***CeuE-Ni@S. One h at 45 °C, pH 7 and 800 rpm. Disassembly was carried out before cycle 24 using sodium dithionite. Reassembly was carried out before cycle 25. *(R)-***2** e.e. checked at selected cycles: 38 ± 5% (1), 36 ± 2% (2), 39 ± 5% (23), 29 ± 8% (25), 33 ± 5% (26), and 44 ± 1% (34). Substrate concentration: 2 mM. Catalyst: 25 μM (1.25 mol %). Catalytic buffer: 0.6 M MES/3 M HCOONa. Error bars show the mean absolute deviation. Accumulated TON 215 (first 7 cycles), 520 (first 24 cycles). TOF 0.51 min^–1^ (average of 7 first cycles).

To assess the prolonged stability of [Fe^III^(**3**)Cp*Ir^III^] ⊂ 6His-***Gst***CeuE-Ni@S and obtain proof of concept for the disassembly and reassembly of the ATHase while the scaffold is immobilized, the immobilized ArM was subjected to several sequential catalytic reaction cycles (Figure 4). While for the first four cycles activity was preserved, in subsequent cycles a gradual reduction of activity was evident, decreasing to around 40% of the initial activity after cycle 23. To investigate whether the ArM leaking from the resin may be responsible for the decrease in relative yield, the separated product mixtures were analyzed for protein content using Bradford’s assay; however, no protein was detected.^48^ To check if the catalytic cofactor leaked from the immobilized ArM, the concentration of Ir in the separated product mixtures was analyzed by ICP MS. The amount of Ir detected in the combined eluents (after 10 recycling cycles) represented only 0.05% of the Ir present in the immobilized ArM prior to catalysis (Table S2). The amount of Ir adsorbed on the frits used to filter the resin beads after each cycle (Figure 2) was also investigated by ICP MS and remained below the limit of quantification. Taken together, the ICP-MS measurements suggest that only a very small proportion of Ir leaked from [Fe^III^(**3**)Cp*Ir^III^] ⊂ 6His-***Gst***CeuE-Ni@S during catalysis. The Fe concentrations were less informative since the unmodified Ni@S beads have a relatively high Fe content, even before the immobilization of the ArM. It is therefore unclear whether the Fe found in the eluent originated from the immobilized ArM or the Ni@S beads themselves.

Since no protein and very little Ir were detectable in the eluents, it was concluded that the catalyst had lost most of its activity. Therefore, the now deactivated catalyst was reductively released from the protein scaffold, *via* reduction of the Fe^III^ to Fe^II^ with sodium dithionite (see supplementary methods). The reduction of Fe^III^ to Fe^II^ was confirmed by using ferrozine as an indicator. The Fe^II^ complex formed with ferrozine shows an intense absorption band at around 560 nm (Figure S5). The complete reduction of Fe^III^ proved challenging even under an inert atmosphere, and some residual catalytic activity remained after the reduction step, cycle 24.

Nonetheless, the immobilized scaffold was recharged with fresh [Fe^III^(**3**)Cp*Ir^III^], and further catalytic reaction cycles were performed. The original activity of the ArM was completely restored in cycle 25, and subsequently, [Fe^III^(**3**)Cp*Ir^III^] ⊂ 6His-***Gst***CeuE-Ni@S was used for another nine cycles. The decrease in activity for the second round (cycles 25–34) was more pronounced than that seen in the first round (1–10). This may be attributed to traces of unbound cofactor retained after system recharging (vide infra, Figure S6). In particular, the recharging process was conducted with the scaffold immobilized, whereas in the primary set, the ArM assembly occurred prior to immobilization. We propose that mass transfer limitations may have impeded the complete assembly of the ATHase during the recharging process, and the complete removal of unbound and nonspecifically adsorbed cofactor, which requires extensive resin resuspension and washing, proved challenging. In contrast, the enantioselectivity was preserved, as verified by chiral HPLC analysis performed after cycles 1, 2, 23, 25, 26, and 34.

### Immobilization and Catalytic Investigation of ATHases Prepared Directly from Cell Lysates

**Figure 5:**
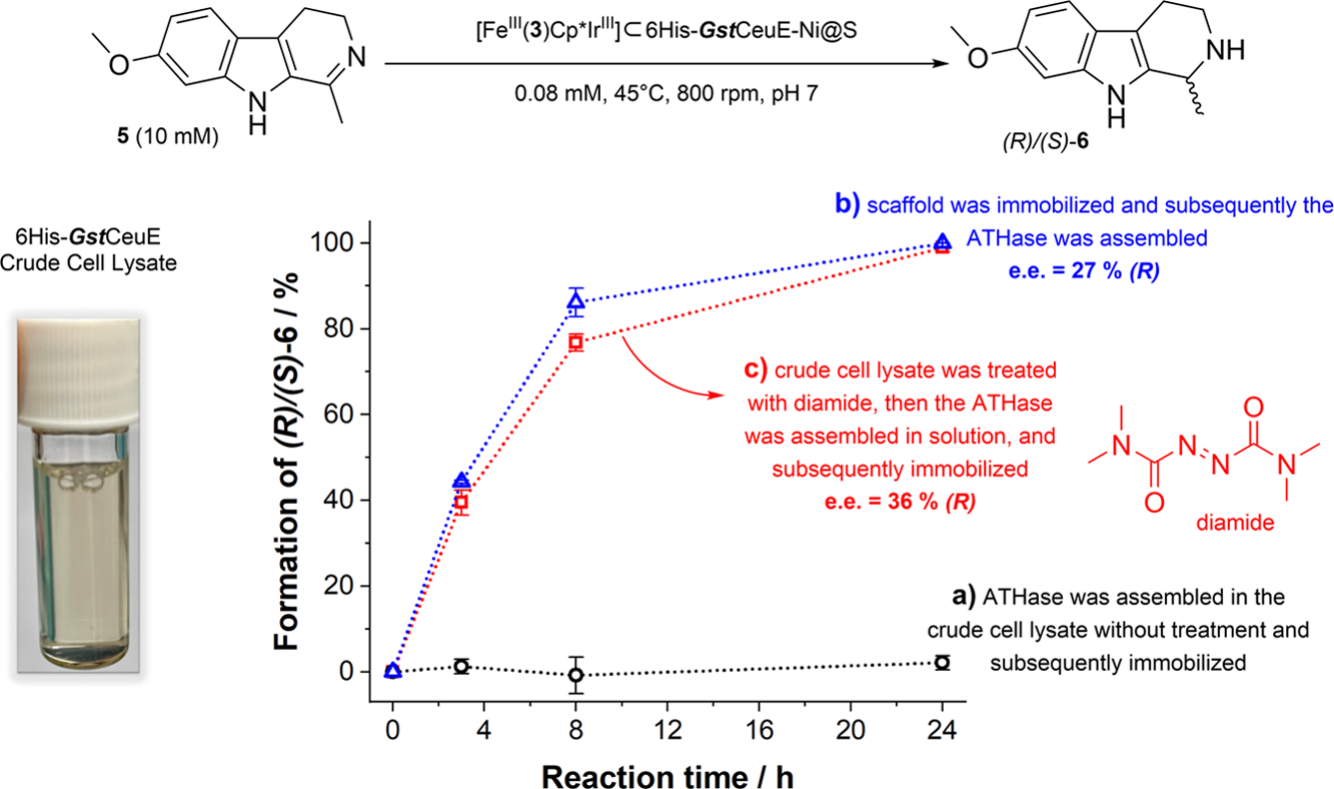
ATHase assembly and immobilization from crude cell lysates. Kinetic profiles were obtained using the immobilized ATHases-Ni@S prepared with 6His-***Gst***CeuE crude cell lysate via approaches **a**, **b** and **c**, for the reduction of **5**.

To avoid costly protein purification steps and facilitate genetic optimization, the 6His-tagged ATHase [Fe^III^(**3**)Cp*Ir^III^] ⊂ ***Gst***CeuE was assembled directly from cell lysates (Figure 5). The chromophoric substrate harmaline (**5**) was chosen to allow initial reactivity tests to be assessed by monitoring the color change from yellow to colorless. Due to the lower solubility of **5** in the catalytic buffer, the reaction conditions had to be slightly adjusted. Initial attempts failed due to cofactor poisoning (Figure 5a), mainly related to the high concentration of glutathione commonly found in the lysates.^7,49−51^ Subsequently, glutathione effects were minimized in two different ways: (1) ATHase assembly after scaffold immobilization (Figure 5b), where glutathione and impurities were removed by washing before the cofactor was introduced, and (2) addition of tetramethylazodicarboxamide (diamide, Figure 5c) as an oxidizing agent. The latter was inspired by the studies reported by Wilson et al.^51^ in which diamide was shown to be effective in preventing iridium catalyst poisoning in cell lysates. Both approaches proved to be effective in circumventing cofactor inhibition during ATHase assembly from the cell lysate. Regardless of the assembly approach, [Fe^III^(**3**)Cp*Ir^III^] ⊂ 6His-***Gst***CeuE-Ni@S completed the reduction of **5** within 24 h, but cofactor binding after scaffold immobilization resulted in a slightly lowered e.e. of 27%. This decrease may be due to the aforementioned partial unspecific binding of the cofactor to the Ni@S resin. A degree of unspecific binding of the cofactor was seen in a control experiment in which Ni@S was mixed with [Fe^III^(**3**)Cp*Ir^III^] instead of the His-tagged ATHase (Figure S6). In contrast, ATHase assembly before immobilization achieved an e.e. of 36%, which is in line with that achieved with purified protein. By being able to “catch” the protein scaffold directly from crude cell extracts and then using the immobilized scaffold for the assembly of the ATHase on the resin, time-consuming protein purification steps can be avoided, and access to an expensive protein purification system is not required. Hence, our approach facilitates the catalytic screening and the optimization of ArMs, and with that their tailoring to specific catalytic applications.

## Summary and Conclusions

In summary, two new redox-reversible ATHases were developed using scaffolds from thermophilic bacteria (***Gst***CeuE and ***Pth***CeuE) in combination with an Fe^III^-siderophore-iridium catalyst, [Fe^III^(**3**)Cp*Ir^III^]. Imine reduction rates around 3.5 times faster than that of our previously reported ATHase, [Fe^III^(**3**)Cp*Ir^III^] ⊂ ***Cj***CeuE, were achieved, albeit at the cost of a slight erosion in enantioselectivity. Both [Fe^III^(**3**)Cp*Ir^III^] ⊂ ***Gst***CeuE and [Fe^III^(**3**)Cp*Ir^III^] ⊂ ***Pth***CeuE gave rise to TOFs (1.71 min^–1^) and e.e. values (29 and 24%, respectively) that are similar to other ATHases that have not undergone genetic optimization. The seminal biotin-anchored Artificial Hydrogenase reported by Collot et al.^52^, e.g., showed an initial e.e. of 37% and required 15 h to achieve 90% conversion at temperature for the reduction of acetamido acrylic acid prior to genetic optimization of the avidin protein scaffold. The subsequent development of a sulfonamide-anchored ATHase by Heinisch et al.^53^ started with an e.e. of 58%, a TON of 25, and a reaction time of 18 h at room temperature for the production of (*S*)-salsolidine using a wild-type hCAII protein scaffold. Both ArMs achieved e.e. values of >95% once optimized. We are therefore optimistic that the genetic optimization of our ATHase will be possible.

To capitalize on our “catch-and-release” approach to ArM design and facilitate the genetic optimization of ATHases by enabling on-resin assembly and activity screening of variants, [Fe^III^(**3**)Cp*Ir^III^] ⊂ 6His-***Gst***CeuE was immobilized on commercially available Ni-NTA Sepharose beads (Ni@S). Upon immobilization, enantioselectivity was retained while the stability of the ATHases was improved, which allowed several recycling cycles. Most importantly, once a significant drop in catalytic activity was observed after 23 consecutive reaction cycles, the inactivated [Fe^III^(**3**)Cp*Ir^III^] cofactor could be removed via reduction of Fe^III^ to Fe^II^, and the immobilized scaffold recharged with active [Fe^III^(**3**)Cp*Ir^III^]. Recharging restored the ATHase’s initial catalytic activity and permitted reuse in further reaction cycles. Importantly, [Fe^III^(**3**)Cp*Ir^III^] ⊂ 6His-***Gst***CeuE-Ni@S could be prepared directly from crude cell extracts and applied to the conversion of **5** to (*R*)/(*S*)-**6**, thereby avoiding time-consuming and expensive protein-purification steps. Capturing generated protein scaffolds on IMAC resins represents a key step in our quest for a high-throughput screening method that will enable us to assess protein variants obtained via either targeted mutagenesis, in particular of amino acid residues near the catalytically active iridium center or within the flexible His-containing loop, or iterative random mutagenesis at more distant positions.

Overall, this investigation demonstrates that the combination of reversible artificial cofactor anchoring with ArM immobilization can significantly increase the reusability of ArMs and their components, thereby improving their applicability, cost-efficiency, and environmental footprint. We envisage the translation of the catch-and-release approach into flow-chemistry applications, e.g., to facilitate catalyst discovery and substrate screening. Furthermore, the demonstrated assembly of ATHase directly from cell lysate can be explored to generate a high-throughput screening platform to directly test mutants and improve the enantioselectivity of ATHases using the unique “catch-and-release” features.

## Methods

### General Artificial Transfer Hydrogenases Preparation

6His-tagged [Fe^III^(**3**)Cp*Ir^III^] ⊂ 6His-***Cj***CeuE, [Fe^III^(**3**)Cp*Ir^III^] ⊂ 6His-***Gst***CeuE, and [Fe^III^(**3**)Cp*Ir^III^] ⊂ 6His-***Pth***CeuE and untagged [Fe^III^(**3**)Cp*Ir^III^] ⊂ ***Cj***CeuE, [Fe^III^(**3**)Cp*Ir^III^] ⊂ ***Gst***CeuE, and [Fe^III^(**3**)Cp*Ir^III^] ⊂ ***Pth***CeuE ATHases were prepared by adapting our previously published procedure.^2^ First, protein stocks were prepared in 0.05 M Tris–HCl/0.15 M NaCl buffer (pH 7.5) (binding buffer), and the concentration determined using the absorbance at 280 nm and the following corrected theoretical molar extinction coefficients: ε_***Cj***CeuE_ = 18590 cm^–1^ M^–1^, ε_***Pth***CeuE_ = 29196 cm^–1^ M^–1^ and ε_***Gst***CeuE_ = 34239 cm^–1^ M^–1^.^45^ [Fe^III^(**3**)Cp*Ir^III^] was produced by adding 75 μL of a 10 mM Fe^III^–NTA solution (as previously described^54^) to 120 μL of 0.2 M MOPS/0.15 M NaCl buffer (pH 7.5) and incubation in a sonication bath for 1 min. Subsequently, 75 μL of **3** (10 mM in DMF) was added, and the mixture was sonicated for 1 min. Then, 75 μL of **4** (5 mM in DMF) was added, and the mixture was sonicated for 1 min. The respective artificial transfer hydrogenases were formed by mixing 400 nmol of protein stock with an equivalent amount of [Fe^III^(**3**)Cp*Ir^III^], and the volume of the resulting solution was made up to 15 mL with binding buffer. The resulting solutions were stored at <8 °C overnight followed by spin concentration using a Vivaspin 20 centrifugation filter (10 kDa molecular weight cut off) at 4500 rpm, 15 min. Concentrated samples were resuspended in binding buffer and centrifuged for another three cycles to wash out any unbound ligand. See the Supporting Information for information on [Fe^III^(**3**)Cp*Ir^III^] ⊂ 6His-***Gst***CeuE preparation from crude cell extracts.

### General Immobilization Protocol

One milliliter of Ni Sepharose 6 Fast Flow 50% resin slurry (Ni@S) was packed into a Mono Q 5/50 column (Cytiva, USA) and mounted as illustrated in Figure 2a. The top adaptor was then connected to a reservoir, and the bottom adaptor was connected to an empty syringe. After equilibration with 5 mL binding buffer, 400 nmol of [Fe^III^(**3**)Cp*Ir^III^] ⊂ 6His-***Gst***CeuE—or alternative tagged ATHases—were added to the reservoir and slowly passed through the resin (∼0.5 mL/min) with the help of the syringe at the bottom. The resin was washed with 10 mL of binding buffer to remove any unbound ArM. Next, 5 mL of storage buffer (0.05 M MES/0.25 M HCOONa/pH 7.0) was passed through to equilibrate the immobilized ArM for storage and future catalytic applications. The immobilization yield was monitored via UV–vis spectroscopy and confirmed using Bradford’s method.^48^ Finally, the immobilized ATHases ([Fe^III^(**3**)Cp*Ir^III^] ⊂ 6His-***Gst***CeuE-Ni@S, [Fe^III^(**3**)Cp*Ir^III^] ⊂ 6His-***Pth***CeuE, and [Fe^III^(**3**)Cp*Ir^III^] ⊂ 6His-***Cj***CeuE) were unpacked into glass vials and the concentration adjusted to 250 μM with storage buffer for future use in batch reactions. See the Supporting Information for information with regard to [Fe^III^(**3**)Cp*Ir^III^] ⊂ 6His-***Gst***CeuE-Ni@S preparation from crude cell extracts.

### General Batch Reaction Procedure

His SpinTrap (GE Healthcare, USA) purification columns were adapted for batch reactions in a thermoshaker for microtubes (Grant-bio). Prior to first use, the original content was removed, and the cartridges were washed thoroughly with distilled water. Then, 50 μL of 250 μM immobilized ArM suspension was poured into a precleaned His SpinTrap cartridge, and storage solvent was removed by centrifugation (500×*g*, 1 min). The bottom outlet was sealed, 450 μL of catalytic buffer was added, and the vial was transferred to a shaker for preconditioning at the desired temperature for 5 min. In sequence, 50 μL of 20 mM 6,7-dimethoxy-1-methyl-3,4-dihydroisoquinoline (**1**) in catalytic buffer was added, the vial was capped and shaking immediately started. The pH, shaking, and temperature effects were investigated. Recyclability and redox-reversible assembly studies were also carried out in these adapted reactors. Figure 2b summarizes the adapted setup, which provided a straightforward way of separating the immobilized ATHases from the reaction medium and facilitated recycling by centrifugation (500×*g*, 1 min). Further details can be found in the supplementary methods, including the homogeneous catalytic reactions, the sample preparation for analytical measurements, and the redox release and recharging of the scaffold for recyclability tests.

## Supporting Information Available

General information, additional experimental methods, supplementary figures and tables, as well as characterization and analytical data (UV–vis, HPLC, ^1^H and ^13^C NMR and ESI) (PDF)
