# Supplementary material for: Catch-and-Release: The Assembly, Immobilization, and Recycling of Redox-Reversible Artificial Metalloenzymes
Source: ACS Catal. 2024 Feb 15;14(5):3218–27. doi: 10.1021/acscatal.3c05294 (PMC10913039; doi:10.1021/acscatal.3c05294)
Supplement: Supplementary file 1 — cs3c05294_si_001.pdf [file cs3c05294_si_001.pdf]

Supplementary information

Catch-and-Release: The Assembly, Immobilisation and Recycling of Redox-reversible Artificial Metalloenzymes

*Alex H. Miller[1], Elena V. Blagova[2], Benjamin Large[1], Rosalind L. Booth[1], Keith S. Wilson[2] and Anne-K. Duhme-Klair[1]**

[1]Department of Chemistry, University of York, Heslington, York, YO10 5DD, United Kingdom

[2]Structural Biology Laboratory, Department of Chemistry, University of York, Heslington, York, YO10 5DD, United Kingdom

*anne.duhme-klair@york.ac.uk

Supplementary information

Table of Contents

1. General information
2. Synthesis of compound 3
3. Cloning, expression and purification of 6His-tagged proteins
4. Binding affinity determination by intrinsic fluorescence quenching
5. Catalytic activity testing

5.1. Stock solutions

5.2. Homogeneous batch reactions – controls

5.3. Heterogeneous batch reactions

6. Product analysis

6.1. UV-Vis analysis

6.2. Non-chiral HPLC analysis

6.3. Chiral HPLC analysis

7. Reductive release of the catalyst and reassembly of the immobilised ArM
8. Preparation of $[Fe^{III}(3)Cp^*Ir^{III}]\subset$6His-*Gst*CeuE-Ni@S from crude cell lysate
9. Control reactions for the recyclability tests monitored by ICP-MS
10. Supplementary figures and tables
11. Supplementary references

Supplementary information

1. General information

Materials

Unless otherwise noted, reagents were used as received from commercial suppliers and used as supplied unless otherwise stated. All expression media and buffers were prepared using ddH_2O (purification system, Millipore). Solvents for chromatography were HPLC grade. $[Ir(Cp^*)(Cl)_2]_2$ was prepared as described in the literature.[1] 6,7-Dimethoxy-1-methyl-3,4-dihydroisoquinoline (referred as **1** or isoquinoline) and 1-methyl-7-methoxy-3,4-dihydro-β-carboline (referred as **5** or harmaline) were purchased from Acros and Sigma-Aldrich, respectively.

Instrumentation

^1H and $^{13}C\{^1H\}$ NMR spectra were recorded on Jeol EX and ES 400 MHz instruments (^1H NMR 400 MHz, ^{13}C NMR 101 MHz). Electrospray ionisation mass spectrometry (ESI-MS) was performed on a Bruker compact® TOF mass spectrometer. Fluorescence spectra were recorded on a Hitachi F-4500 fluorescence spectrophotometer. UV-vis spectra were recorded on a Shimadzu UV-1800 in a quartz cuvette (Starna scientific). HPLC measurements were performed on an Agilent 1200 infinity II quaternary system equipped with a 1260 Quaternary Pump G7111B, G7116A multicolumn thermostat, G7165A multiwavelength detector and G7129A Vialsampler using the specified eluent gradients. Inductively coupled plasma-optical emission spectroscopy (ICP-OES) was carried out on an Agilent 5100 spectrometer and analysis was performed in ICP Expert version 7.6.2.12331. (ICP-MS) was carried out on an Agilent 7700 (Biorenewables Development Centre, York, United Kingdom).

Data accessibility

The data supporting this research is available for download from the research data repository of the University of York at https://doi.org/10.15124/85cd4daf-de96-4192-9712-1180496c51f9.

2. **Synthesis of compound 3**

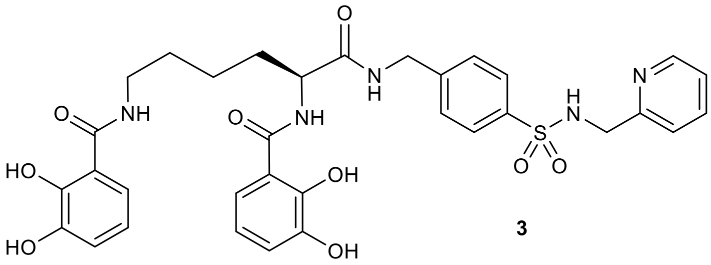

3

3 was synthesised according to a previous published method, with minor adaptations.[2] The glassware used in this reaction was soaked in HCl (6 M), then successively rinsed with deionised H_2O, concentrated NaOH, deionised H_2O and dry EtOH to remove any residual metal ions. Dry EtOH (8 mL) and NH_3 (18 M in MeOH, 2 mL) were added to a 25 mL round bottom flask containing *N*, *N'*-(6-oxo-6-(((4-(N-(pyridin-2-ylmethyl)sulfamoyl)benzyl)amino)oxy)hexane-1,5-diyl)bis(2,3-bis(benzyloxy)benzamide) (100 mg, 96.3 µmol, 1 eq.) and $Pd(OH)_2$ (16 mg) under an atmosphere of N_2, followed by H_2. The mixture was stirred vigorously for 4 h before being filtered through a Whatman glass microfiber filter GF/F. After removal of the solvent *in vacuo*, **3** was obtained as an off-white powder (99%).

^{1}H NMR (400 MHz, METHANOL-D_4) δ 8.34 (d, J = 4.8 Hz, 1H), 7.74 (d, J = 8.1 Hz, 2H), 7.68 (td, J = 7.8, 1.8 Hz, 1H), 7.42 (d, J = 8.1 Hz, 2H), 7.38 – 7.31 (m, 2H), 7.25 – 7.16 (m, 2H), 6.92 (t, J = 8.1 Hz, 2H), 6.70 (dt, J = 9.5, 8.0 Hz, 2H), 4.59 (dd, J = 8.5, 5.7 Hz, 1H), 4.50 – 4.38 (m, 2H), 4.15 (s, 2H), 3.39 (t, J = 7.0 Hz, 2H), 2.07 – 1.79 (m, 3H), 1.68 (p, J = 7.3 Hz, 2H), 1.52 (tq, J = 14.0, 7.5 Hz, 2H), 1.38 – 1.22 (m, 1H).

^{13}C NMR (101 MHz, METHANOL-D_4) δ 174.70, 171.51, 171.13, 158.21, 150.22, 149.72, 149.54, 147.31, 147.21, 145.18, 140.44, 138.74, 128.95, 128.24, 123.94, 123.47, 119.80, 119.60, 119.58, 118.64, 117.10, 116.80, 55.27, 48.87, 43.50, 40.12, 32.66, 30.03, 24.43.

HRMS (ESI) m/z: Calcd for $C_{33}H_{34}N_5O_9S$ [M-H]: 676.2083; Found 676.2075.

The NMR and MS spectra are in agreement with those reported in the literature.[2]

3. Cloning, expression and purification of 6His-tagged proteins

The periplasmic binding protein ***Cj***CeuE (44-330) and its thermostable homologues ***Gst***CeuE protein (39-319) and ***Pth***CeuE protein (37-318) (constructs with the signal peptide removed) were chosen, cloned into Lic-adapted pET 28a vector (YSBLic3C) and purified with the presence and absence of a 6-terminal His-tag, as described recently.[3]

4. Binding affinity determination by intrinsic fluorescence quenching

Fluorescence spectra were recorded on a Hitachi F-4500 fluorescence spectrophotometer with an excitation wavelength of 280 nm, emission range of 295-410 nm, 10 nm excitation slit width, 20 nm emission slit width, 60 nm/min scanning speed, automatic response, corrected spectra and 950 V detector voltage. Preparations and measurements were carried out following the procedures previously reported.[3] The only change was that the [Fe^{III}(**3**)Cp*Ir^{III}] stock solution was prepared in DMF to improve solubility (avoid precipitation) and hence the accuracy of quantities being dispensed by DOSTAL DOSY.

5. Catalytic activity testing

5.1. Stock solutions

MES/formate buffer ('catalytic' buffer): The buffer solution was prepared by dissolving 17.06 g of 2-morpholin-4-ylethanesulfonic acid monohydrate (MES monohydrate) and 27.20 g of sodium formate (HCOONa) in 80 ml water and then adjusting the volume to 100 mL (final concentration: 0.8 M MES, 4 M HCOONa). 7.5 mL of the stock solution were transferred to a flask and the pH adjusted to either 6, 6.5. 7, 7.5 or 8 by addition of 5 M NaOH before the solution volume was brought up to 10 ml (final concentrations 0.6 M MES, 3 M sodium formate).

Isoquinoline stock: 200 or 20 mM stock solutions of isoquinoline (**1**) were prepared in the catalytic buffer of choice.

Harmaline stock: 12 mM stocks of harmaline (**5**) were prepared in the catalytic buffer pH 7.

Quenching solution: 12.5 mM solution of L-glutathione in Water:MeOH (1:2) mixture was prepared, volumes as required. L-Glutathione was firstly dissolved in water before addition of MeOH. Quenching solutions were stored at 4 °C and used on the day of preparation.

5.2. Homogeneous batch reactions – controls

Reactions were carried out analogously to those previously reported,[2] however using a thermos-shaker for microtubes (Grant-bio) instead of magnetic stirrer. For isoquinoline the reactions were run in catalytic buffer at pH 6 (0.6 M MES / 3 M HCOONa), a 50 mM substrate concentration and 0.125 mM catalyst/ArM ([Fe^{III}(**3**)Cp*Ir^{III}], [Fe^{III}(**3**)Cp*Ir^{III}]⊂***Cj***CeuE, [Fe^{III}(**3**)Cp*Ir^{III}]⊂***Gst***CeuE and [Fe^{III}(**3**)Cp*Ir^{III}]⊂***Pth***CeuE) concentration at 40 °C and shaking speed of 400 rpm. Total volume 0.5 mL. At defined time points, 25 µL aliquots were withdrawn from the reaction mixtures and mixed with 1975 µL of the quenching solution. Total volume 0.5 mL. At defined time points, 25 µL aliquots were withdrawn from the reactions and mixed with 1975 µL of the quenching solution. Quenched samples were submitted for UV-vis or HPLC analysis (methods detailed in the next section).

5.3. Heterogeneous batch reactions

Generally, batch reactions with the immobilised ArMs were carried out by using adapted batch reactors, following the 'General Batch Reaction Procedure' provided in the main text. For catalytic tests with isoquinoline as the substrate, a substrate concentration 2 mM was used, unless stated otherwise. Once completed, reaction mixtures were separated from the immobilised ArMs *via* spin filtration. 100 µL aliquots were withdrawn from each collection vials and mixed with 900 µL of quenching solution. For catalytic tests with harmaline, a substrate concentration of 10 mM was used, unless stated otherwise. Upon completion of the reaction, reaction mixtures were separated from the immobilised ArMs *via* spin filtration. 25 µL aliquots were withdrawn from each collection vial and mixed with 1975 µL of quenching solution. Quenched samples were submitted for UV-vis or HPLC analysis (methods detailed in the next section).

For the thermostability assay, immobilised ArMs were incubated in the catalytic buffer at 60 °C, under static conditions, for 4, 8 or 18 h, before the catalytic test was started with the addition

of substrate and start shaking at 800 rpm for the duration of 1 h. A control with no pre-incubation was carried out and used as reference for relative activity calculations.

6. Product analysis

The progress of the imine reduction reactions was monitored by UV/vis spectroscopy *via* the decrease of the absorption bands with maxima at around 350 nm (isoquinoline) and 375 nm (harmaline). UV/vis was found to be sufficient for monitoring reaction evolution, with analogous analytical accuracy when compared to HPLC (**Figure S7**).

For isoquinoline, reaction progress was also monitored by reverse-phase non-chiral HPLC (Athena C18-WP column, 100 Å, 4.6 x 25 mm, CNW), with caffeine as internal standard. Enantiomeric excesses were estimated from reverse-phase chiral HPLC traces (Phenomenex Lux Cellulose-4 column, 250 mm × 4.6 mm, 5 μm). Before HPLC analysis, samples were filtered through 0.22 μm nylon membranes. Methods are detailed in sections 6.1-6.3. Representative non-chiral and chiral chromatograms are presented in **Figures S9** and **S10**, respectively.

6.1. UV-Vis analysis

After quenching, the respective reaction aliquots were transferred to a quartz cuvette and spectra recorded from 500 to 250 nm, 1 nm interval, fast scan.

6.2. Non-chiral HPLC analysis

Non-chiral HPLC method for isoquinoline and (*R*)-(+)/(*S*)-(−)-salsolidine:

Solvent A: H_2O + 0.1% TFA

Solvent B: MeOH + 0.1% TFA

Injection volume: 20 µL

The starting ratio of 90:10 (A:B) and ramped to 50:50 (A:B) over 20 minutes, then ramped to 10:90 (A:B) over 0.5 minutes and held for 3.5 minutes. At 24.5 minutes, the solvent was ramped back to 90:10 (A:B) over 0.5 min and held for 10 minutes. 1 mL min^{-1}, 35 °C, retention times: (*R*)-(+)/(*S*)-(−)-salsolidine 9.8 minutes, isoquinoline 12.0 minutes, caffeine 13.9 minutes.

6.3. Chiral HPLC analysis

Chiral HPLC method for compounds (*R*)-(+)-salsolidine and (*S*)-(−)-salsolidine:

Solvent A: 20 mM $(NH_4)HCO_3$ in HPLC grade H_2O, pH 8.75 adjusted with DEA

Solvent B: Acetonitrile

Injection volume: 20 µL

The starting ratio of 90:10 (A:B) was ramped to 87:13 (A:B) over 12 minutes, then ramped to 60:40 (A:B) over 8 minutes, ramped back to 90:10 (A:B) over 5 minutes and held for 5 minutes. 1.5 mL min^{-1}, 35 °C, retention times: (*S*)-(−)-salsolidine 12.6 minutes, (*R*)-(+)-salsolidine 13.7 minutes.

The absolute configuration of the reduction products (*R*)-(+)-salsolidine and (*S*)-(−)-salsolidine were determined using available commercial standards.

Chiral HPLC method for compounds (*R*)-(+)-tetrahydroharmine and (*S*)-(−)-tetrahydroharmine:

Solvent A: 20 mM $(NH_4)HCO_3$ in HPLC grade H_2O, pH 8.75 adjusted with DEA

Solvent B: Acetonitrile

Injection volume: 10 µL

The starting ratio of 75:25 (A:B)was ramped to 60:40 (A:B) over 15 minutes, then ramped back to 75:25 (A:B) over 0.5 minute and held for 9.5 minutes. 1.0 mL min^{-1}, 35 °C, retention times: (*S*)-(−)-tetrahydroharmine 7.9 minutes and (*R*)-(+)-tetrahydroharmine 8.4 minutes.

The absolute configuration of the reduction products (*R*)-(+)-tetrahydroharmine and (*S*)-(−)-tetrahydroharmine were determined by comparison with samples obtained by reduction using the homogeneous RuCl(p-cymene)[(R,R)-Ts-DPEN] catalyst which affords preferentially the (*S*)-reduction product.[4, 5]

7. Reductive release of the catalyst and reassembly of the immobilised ArM

After cycle 23rd of the recyclability studies (Figure 5), the immobilised ArM ([Fe^{III}(**3**)Cp*Ir^{III}]⊂6His-***Gst***CeuE-Ni@S) was separated from the reaction medium, rinsed with storage buffer (0.05 M MES / 0.25 M HCOONa / pH 7). Any residual buffer was removed via spin filtration for 1 min at 500 *x g*. The reduction-triggered release of the siderophore-catalyst was then performed in a wet glove box to improve reduction yields and avoid Fe^{II} re-oxidation.

General protocol for the reductive release of the catalyst: Aqueous stock solutions of $Na_2S_2O_4$ (200 mg/mL) and ferrozine (3.6 mg/mL) were prepared in ddH_2O. Next, 2 mL of a reduction solution was prepared by mixing 1920 µL buffer (0.1 M MES / 0.5 M NaCl / pH 7.5) with 60 µL ferrozine stock and 20 µL $Na_2S_2O_4$ stock. Then, the bottom outlet of the adapted batch reactor was connected via adaptor to an empty syringe, and the reducing solution was pulled through the immobilisate in 4 steps of 500 µL (all eluates were collected and transferred to a storage vial for analysis). Then, the resin with the immobilised apo-scaffold was rinsed with water. All components were taken out of the glove box for analysis.

After removal of excess sodium dithionite from the eluates by purging with air, the intense magenta colour of the Fe^{II}-ferrozine complex was evident, confirming the successful reduction of Fe^{III} to Fe^{II} (UV-vis scan **Figure S5**). Additionally, the colour of the resin beads changed from purple to blue/green. The recovered immobilised apo-scaffold was then rinsed with storage buffer, and reaction cycle 24th carried out as control. Then, the scaffold was recharged with fresh [Fe^{III}(**3**)Cp*Ir^{III}] as follows. Firstly, the resin with the immobilised apo-scaffold was washed twice with binding buffer (0.05 M Tris-HCl / 0.15 M NaCl / pH 7.5). Then, one equivalent of [Fe^{III}(**3**)Cp*Ir^{III}] (molar equivalent to the amount of immobilised scaffold) was

prepared in binding buffer, total volume 0.5 mL, and added to the reactor containing the immobilised apo-scaffold. The mixture was shaken for 1 h at 800 rpm, 20 °C, and then left under static condition overnight at 4 °C. On the following day, the system was centrifuged for 1 min, 500 *x g*, and the recharged system was rinsed with storage buffer before subsequent reaction cycles were started (25th and further cycles).

8. Preparation of [Fe^{III}(3)Cp*Ir^{III}]⊂6His-*Gst*CeuE-Ni@S from crude cell lysate

Mono Q™ 5/50 columns (Cytiva, USA) were packed with 0.5 mL of Ni Sepharose 6 Fast Flow 50 % resin slurry (Ni@S). The bottom adaptor was connected to an empty syringe and column left uncapped. The resin was then equilibrated with 10 mL binding buffer (0.05 mM Tris-HCl / 0.15 M NaCl / pH 7.5). Fresh cell lysate containing 6His-***Gst***CeuE was obtained in 0.05 M Tris-HCl / 0.5 M NaCl / pH 7.5 and used immediately. Three different preparation procedures were tested, **a**, **b** and **c**, as detailed below:

a: 6 mL of untreated crude cell lysate were stirred for 10 minutes. Then, 200 µL of a 2.174 mM solution of [Fe^{III}(**3**)Cp*Ir^{III}] (prepared as described in the methods section in the main text) was added to assemble the [Fe^{III}(3)Cp*Ir^{III}]⊂6His-***Gst***CeuE ArM and the mixture stirred for another 20 minutes. Next, the mixture went through the immobilisation procedure (described in the methods section in the main text) using the pre-packed Ni@S column. The immobilised ArM washed with 15 mL of binding buffer to remove any impurities and weakly-bound components, and finally rinsed with 5 mL of storage buffer (0.05 M MES / 0.25 M HCOONa / pH 7).

b: 6 mL of untreated crude cell lysate was stirred for 10 minutes. Then, the lysate went through the immobilisation procedure (as described in the methods section in the main text) using the pre-packed Ni@S column. The immobilised 6His-***Gst***CeuE scaffold was washed with 15 mL of binding buffer to remove impurities and weakly-bound components. Next, 200 µL of a 2.174 mM solution of [Fe^{III}(**3**)Cp*Ir^{III}] (prepared as described in the methods section in the main text) was passed through the column, and the resin was washed with 15 mL of binding buffer, followed by 5 mL of storage buffer.

c: 600 µL of a 100 mM solution of tetramethylazodicarboxamide (diamide) in 0.6 M MES / 3 M HCOONa / pH 7 was added to 6 mL of the crude cell lysate (resulting in a solution of ~10 mM diamide) and the resulting mixture was stirred for 10 minutes. Then, 200 µL of a 2.174 mM [Fe^{III}(**3**)Cp*Ir^{III}] solution in (prepared as described in the methods section in the main text) was added and the mixture stirred for another 20 minutes before the mixture was subjected to the immobilisation procedure (as described in the methods section in the main text), using a pre-packed Ni@S column. The immobilised ArM washed with 15 mL of binding buffer to remove any impurities and weakly-bound components, and finally rinsed with 5 mL of storage buffer (0.05 M MES / 0.25 M HCOONa / pH 7).

The respective ArM-Ni@S were unpacked and final suspension volumes adjusted to 3 mL with storage buffer. Next, 1.5 mL of samples **a**, **b** and **c** were transferred to empty His SpinTrap (GE Healthcare) purification columns. The storage buffer was removed by spin filtration, 500 *x g*, 1 min. Then 3 mL (6 x 0.5 mL) of a 500 mM solution of imidazole in binding buffer was passed through to elute the immobilised ArM. The eluted ArMs were then concentrated in Vivaspin 20 centrifugation filters (10 kDA molecular weight cut off), 4500 rpm, 30 min. Concentrated samples were diluted in 10 mL binding buffer and the process repeated twice, before samples were diluted again in 10 mL storage buffer, and concentrated for 40 min. The final concentrated samples were used to estimate the concentration of immobilised ArMs using Bradford's method using a stock of ***Gst***CeuE with known concentration as calibration standard. In addition, the protein content in the cell lysate, in the immobilisation flow-through, and in the final concentrated eluted samples was checked by SDS-PAGE analysis (**Figure S8**).

The reduction of harmaline (**5**) using immobilised ArMs were carried out following the procedure describe in section 5.3.

9. Control reactions for the recyclability tests monitored by ICP-MS

0.6 M MES / 3 M HCOONa / pH 7 catalytic buffer was prepared in HPLC grade water, and stirred in 20 % w/v Chelex® 100 sodium form, 50-100 mesh (dry) resin (Sigma Aldrich), for 24 h, to remove any Fe or Ir traces. The buffer was separated from Chelex resin via centrifugation and the pH corrected using 5 M NaOH solution after Chelex treatment carried

out in the same fashion. Chelexed buffer was used to prepare a 2 mM isoquinoline stock, and control reactions performed following procedure as described for the recyclability studies in the main text (**Figure 4**). The eluent from 10 consecutive reaction cycles were combined (total 5 mL) and sent to ICP-MS analysis (**Table S2**, entry 3), along with the following controls: aliquots of commercial Ni@S resin (**Table S2**, entry 1), aliquots of [Fe^{III}(3)Cp*Ir^{III}]⊂6His-***Gst***CeuE in storage buffer (**Table S2**, entry 2), recovered polyethylene frits from the His Spin Trap adapted reactors, **Figure 2** (**Table S2**, entry 4), and finally, aliquots of 2 mM isoquinoline stock in chelexed catalytic buffer (**Table S2**, entry 5).

Sample digestion procedure for ICP-MS analysis:

1) Each sample was weighed out and placed into one digestion vessels.

2) 5 mL of concentrated nitric acid, 1 mL of concentrated hydrochloric acid and 1 mL of 30% H_2O_2 were added into the digestion vessels. A 16 digestion vessel was prepared with 5 mL of concentrated nitric acid, 1 mL of concentrated hydrochloric acid and 1 mL of 30% H_2O_2. This vessel served as the blank, which was used to dilute the calibration fluids later.

3) The digestion vessels were sealed and placed into the microwave. A thermocouple was placed into the 1st digestion vessel, in order to monitor the temperature of the liquid inside. The microwave was programmed to heat the contents of the digestion vessels to 200 °C over a period of 30 minutes. Once at temperature, the contents were kept at 200 °C for a period of 15 minutes. After this period, the microwave heating is turned off and the digestion vessels are cooled down.

4) The digestion vessels were removed from the microwave, then the contents were emptied into 100 mL conical flasks and diluted to up to the mark with distilled water. 10 mL of each sample was placed in a 15 mL sterilised centrifuge tube, in preparation for the analysis.

10. Supplementary figures and tables

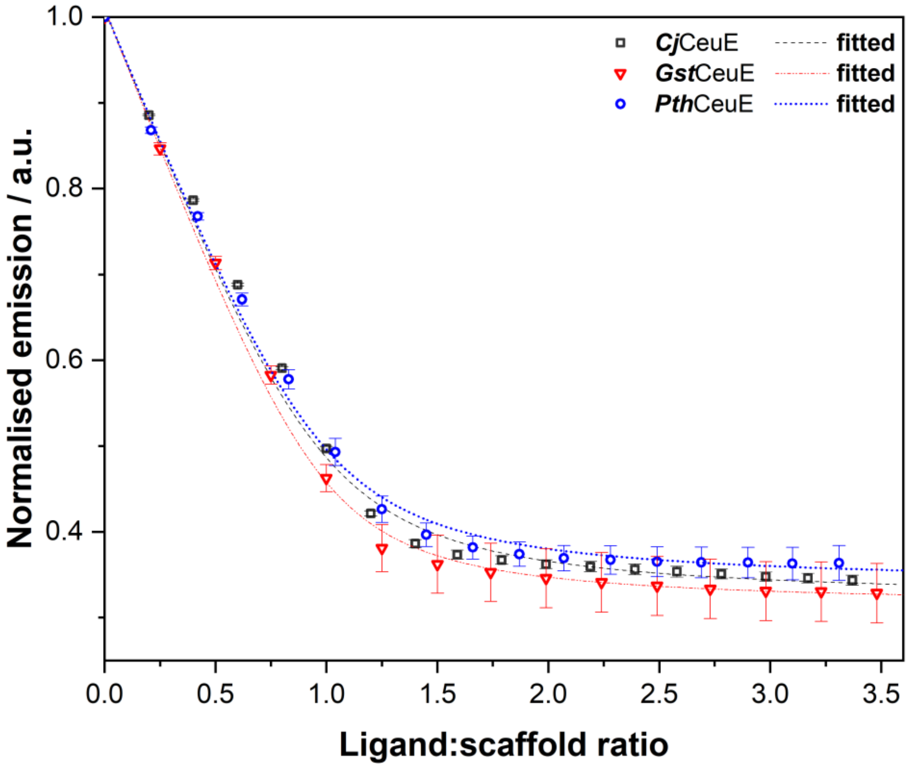

Figure S1. Fluorescence quenching observed on addition of [Fe^{III}(**3**)Cp*Ir^{III}] to ***Cj***CeuE, ***Gst***CeuE and ***Pth***CeuE. Stock solution concentrations as follows: ***Cj***CeuE & [Fe^{III}(**3**)Cp*Ir^{III}] = 300 nM & 120 μM, ***Gst***CeuE & [Fe^{III}(**3**)Cp*Ir^{III}] = 180 nM & 90 μM and ***Pth***CeuE & [Fe^{III}(**3**)Cp*Ir^{III}] = 240 nM & 100 μM.

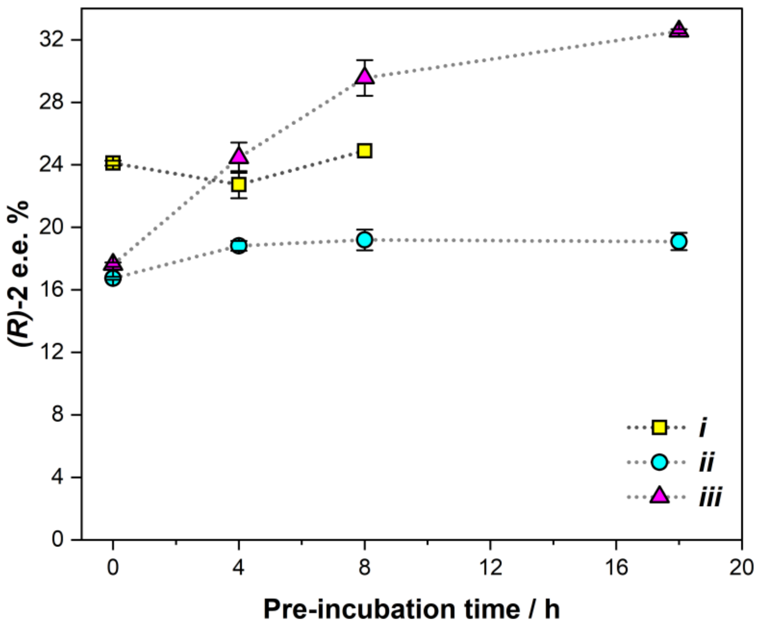

Figure S2. Enantiomeric excess from the reactions catalysed by [Fe^{III}(**3**)Cp*Ir^{III}]⊂6His-***Cj***CeuE-Ni@S (*i*), [Fe^{III}(**3**)Cp*Ir^{III}]⊂6His-***Pth***CeuE-Ni@S (*ii*) and [Fe^{III}(**3**)Cp*Ir^{III}]⊂6His-***Gst***CeuE-Ni@S (*iii*) for the reduction of **1** to *(R)/(S)*-**2** achieved at 60 °C, pH 7, 800 rpm after ATHase pre-incubation at 60 °C for 0, 4, 8 and 18 h. Error bars show the mean absolute deviation. The chiral separation couldn't be verified at 18 h for [Fe^{III}(**3**)Cp*Ir^{III}]⊂6His-***Cj***CeuE-Ni@S due to low yield of conversion, which is below chromatographic detection limit.

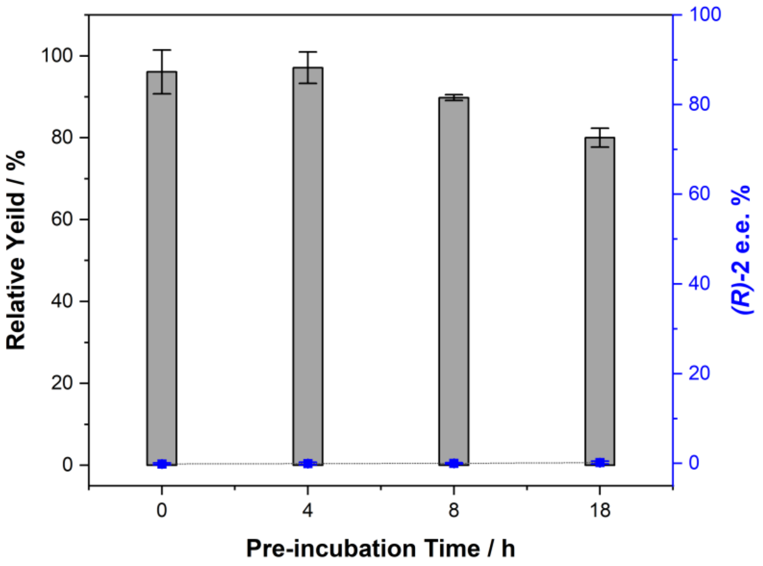

Figure S3. Catalytic performance of [Fe^{III}(**3**)Cp*Ir^{III}] for the reduction of **1** to *(R)/(S)*-**2** achieved at 60 °C, pH 7 and 800 rpm after ATHase pre-incubation at 60 °C for 0, 4, 8 and 18 h (columns). No enantioselectivity observed in all cases (squares). Substrate concentration: 10 mM. Catalyst: 25 µM (0.25 mol%). Catalytic buffer: 0.6 M MES / 3 M HCOONa / pH 7. Error bars show the standard deviation.

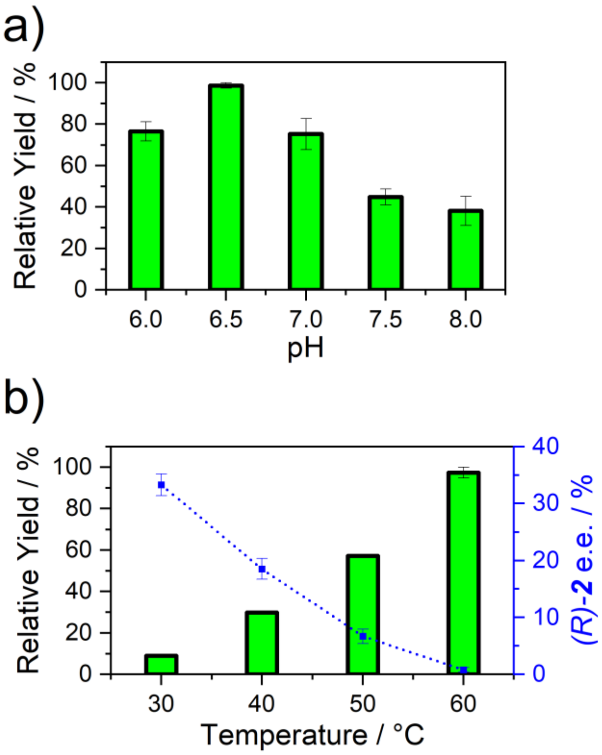

Figure S4. Free untagged [Fe^{III}(**3**)Cp*Ir^{III}]⊂***Gst***CeuE batch reaction conditions survey. a) Catalytic performance as a function of pH at 40 °C, 400 rpm. b) Temperature dependence at pH 6.5, 400 rpm (columns), and respective (*R*)-**2** enantiomeric excesses (symbols + dotted line). Substrate concentration: 5 mM. Catalyst loading: 0.4 mol%. Catalytic buffer: 0.6 M MES / 3 M HCOONa / (pH adjusted with concentrated NaOH). Error bars shows the mean absolute deviation.

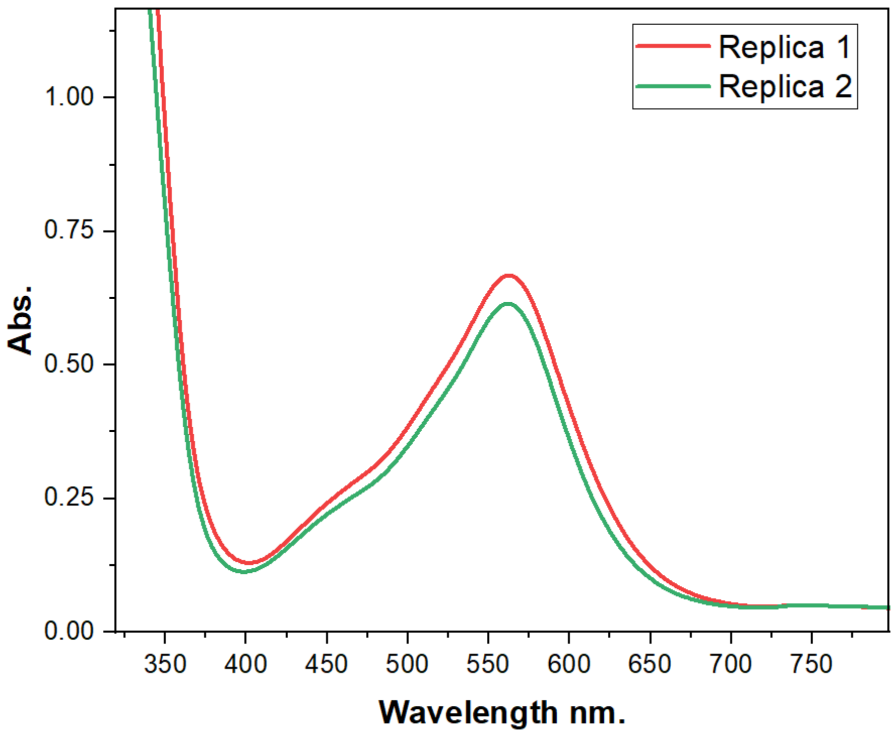

Figure S5. Absorption spectra obtained of the Fe^{II}-ferrozine complex after Fe^{III} reduction**.** Two replicates were subjected to the reductive-release procedure as described, and the presence of Fe^{II} confirmed by chelation with ferrozine, which produces a Fe^{II} complex with characteristic absorbance at around 560 nm.

Table S1. ICP-OES analysis

Entry	Sample Description / Denomination	Fe / nmol	Ir / nmol	Fe/Ir
1	[Fe^{III}(**3**)Cp*Ir^{III}]	111.01	95.61	1.16
2	[Fe^{III}(**3**)Cp*Ir^{III}]⊂6His-***Gst***CeuE	29.85[a]	29.8	1.00
3	[Fe^{III}(**3**)Cp*Ir^{III}]⊂6His-***Gst***CeuE-Ni@S	7.84[b]	7.79[c]	1.00
4	Tris-HCl buffer (0.05 M Tris-HCl / 0.15 M NaCl / pH 7.5)	9.78	<LOQ	-
5	Catalytic Buffer (0.6 M MES / 3 M HCOONa / pH 7)	0.33	<LOQ	-
6	Ni@S	4.12	0.73	5.68

[a] Fe content after subtraction of the Fe background level found in the respective sample buffer (entry 4).

[b] Fe content after subtraction of the Fe background level found in the respective sample buffer (entry 5) and in the unmodified resin (entry 6).

[c] Ir content after subtraction of the Ir background level found in the unmodified resin (entry 6).

<LOQ: below limit of quantification

Table S2. ICP-MS analysis monitoring of Fe and Ir in reaction eluents collated during the recyclability studies.

Entry	Sample Description / Denomination	Raw Data					Processed Data [c]	
		Repetition	Dilution [a]	Sample mass (g) [b]	^{56}Fe [He] / [ppb]	^{193}Ir [He] / [ppb]	nmol ^{56}Fe / g	nmol ^{193}Ir / g
1	Ni@S	1	1236.09	0.0809	13616.7	<LOQ	301368	<LOQ
2	[Fe^{III}(**3**)Cp*Ir^{III}]⊂6His-***Gst***CeuE-Ni@S	1	968.05	0.1033	10266.3	9116.3	202296 ± 34437	45479 ± 607
		2	811.03	0.1233	15607.7	10677.7		
3	Reaction eluents[d]	1	91.86	1.0886	1033.0	47.4	1146 ± 781	24 ± 2
		2	68.12	1.4679	487.2	73.3		
4	Polyethylene Frits	1	2024.29	0.0494	<LOQ	<LOQ	<LOQ	<LOQ
		2	1984.13	0.0504	<LOQ	<LOQ		
5	Substrate stock in catalytic buffer[e]	1	56.17	1.7803	<LOQ	<LOQ	<LOQ	<LOQ

[a] Final dilution of digested samples before analysis (made up to 100 mL).

[b] Weighed sample for digestion. Digestion carried out as described in the respective supplementary method (Supplementary information, section 9)

[c] Calculated Fe and Ir content (nmol) normalised by the weighed sample (g).

[d] Accumulated elution (5 mL) from 10 consecutive reaction cycles.

[e] 2 mM isoquinoline in chelexed catalytic buffer (0.6 M MES/ 3 M HCOONa / pH 7).

<LOQ: below limit of quantification

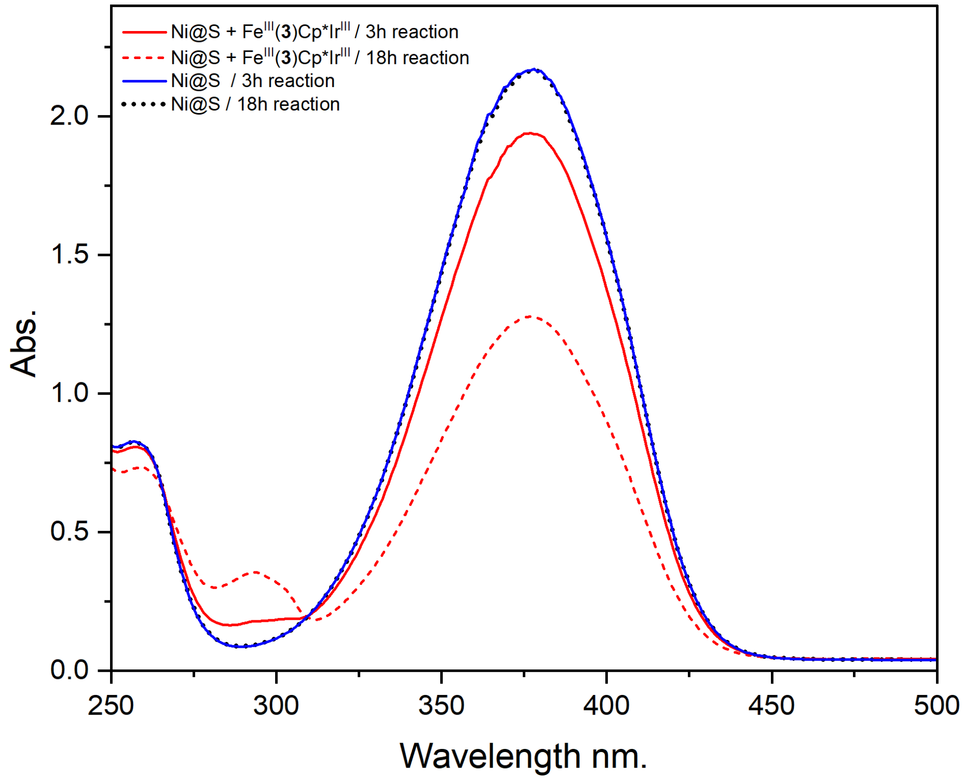

Figure S6. Control reactions to survey unspecific binding of [Fe^{III}(**3**)Cp*Ir^{III}] to the Ni@S resin. Experiments were carried out in which Ni@S was mixed with [Fe^{III}(**3**)Cp*Ir^{III}] instead of the His-tagged ATHase. After being subjected to steps analogous to the the general immobilisation protocol, the recovered resin (consisting of Ni@S and potential traces of unspecific bound [Fe^{III}(**3**)Cp*Ir^{III}]) was tested for harmaline (**5**) reduction. The general reaction procedure is described in section 5.4. The UV-vis spectra of the reaction mixture showed a decrease in the intensity of the absorption band at around 375 nm and the appearance of a band at 290 nm, indicating a degree of product formation and hence unspecifically-bound catalyst. Unmodified bare Ni@S resin was also tested as a negative control, and showed no catalytic activity.

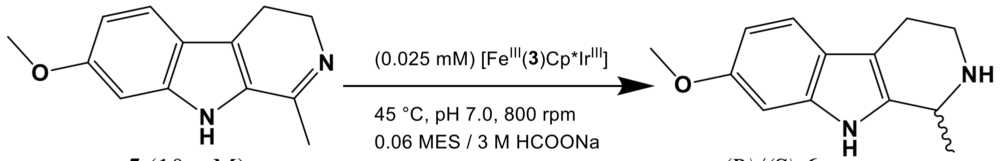

5 (10 mM) *(R)/(S)*-**6**

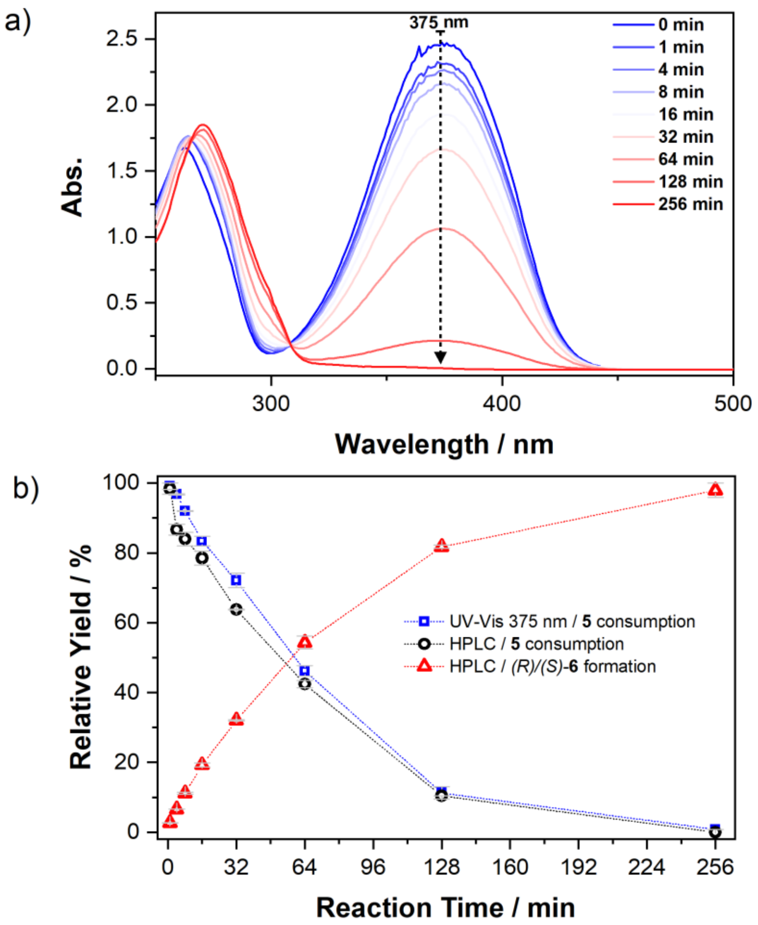

Figure S7. UV-vis spectra and HPLC traces obtained whilst monitoring the progress of the catalysed reduction of 5 (harmaline)**. a)** The decrease in absorbance at 375 nm is indicative of the consumption of the imine substrate (and concomitant amine formation). **b)** Comparison of the decrease in substrate concentration over time, as monitored by UV-vis spectroscopy and HPLC, plus product formation over time detected by HPLC.

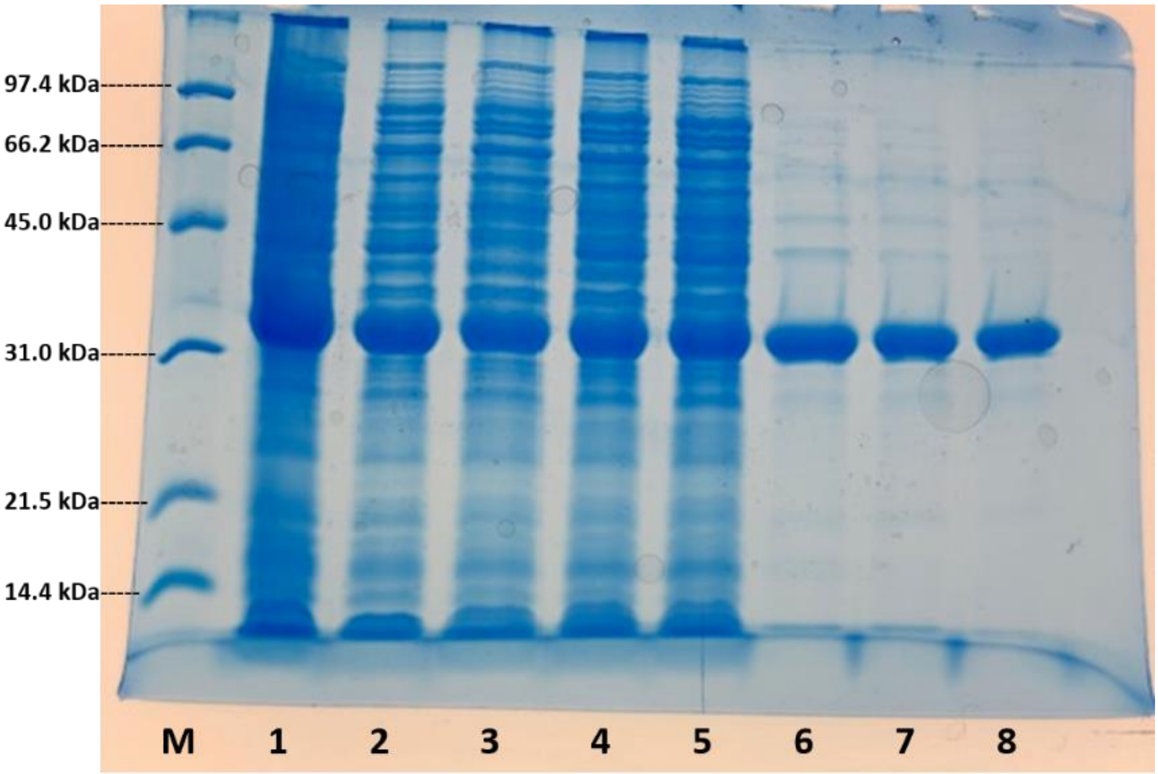

Figure S8. SDS-PAGE of 6His-***Gst***CeuE as part of ArMs preparation directly from cell lysate. Lane M: low molecular weight protein marker, Lane 1: cell lysate, Lane 2: cell lysate 3x dilution, Lane 3: preparation **c** flow-through from immobilisation step, Lane 4: preparation **b** flow-through from immobilisation step, Lane 5: preparation **a** flow-through from immobilisation step, Lane 6: preparation **c** final eluted and concentrated ATHase, Lane 7: preparation **b** final eluted and concentrated ATHase, Lane 6: preparation **a** final eluted and concentrated ATHase.

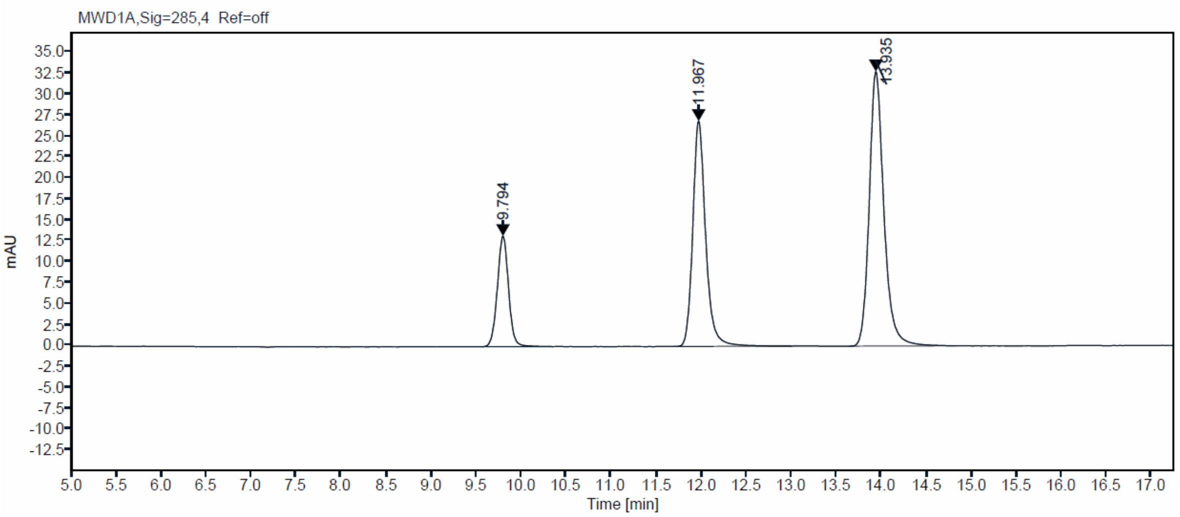

Figure S9. Non-Chiral HPLC separation of substrate (**isoquinoline** – 12.0 min), product (**salsolidine** - 9.8 min) and internal stantdard (**caffeine** - 13.9 min).

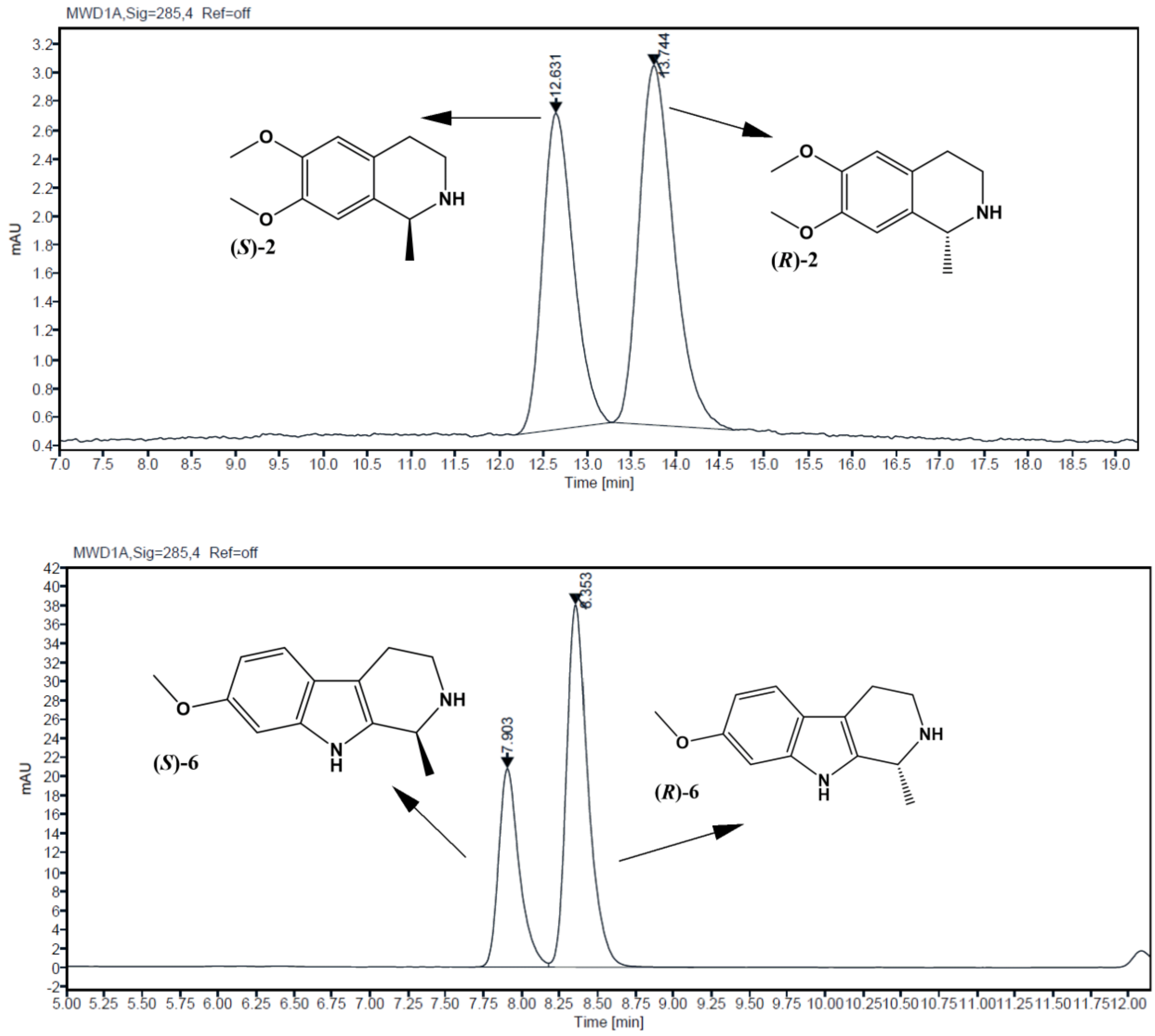

Figure S10. Chiral HPLC separation of products **2** (top) and **6** (bottom).

Analysis Information

Analysis Filename	akdk98024bl_neg_P1-F-3_01_35203.d	Acquisition Date	08/07/2022 15:57:56
Method	ESI_low mass neg_2c1s.m	Instrument	compact
Submission Name	akdk98024bl_neg	ESI	Negative

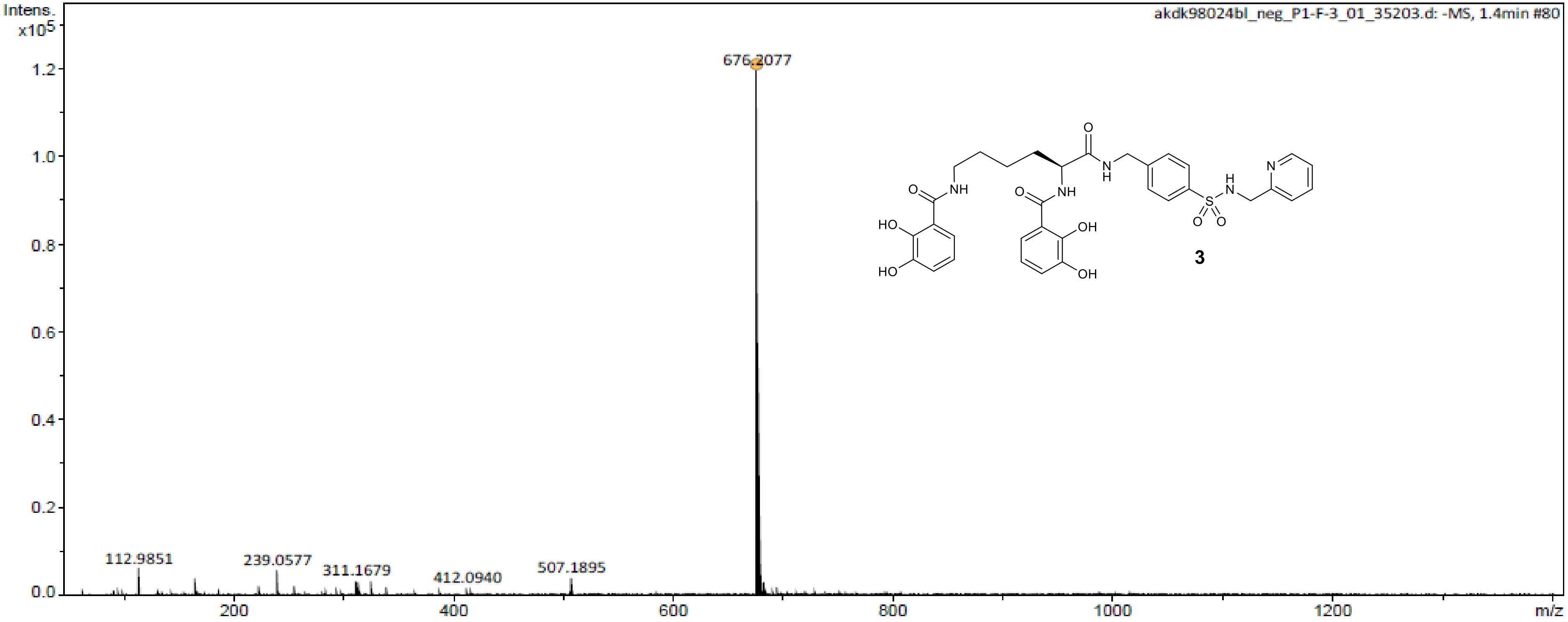

Meas. m/z	#	Ion Formula	m/z	err [ppm]	err [mDa]	mSigma	Mean err [ppm]
676.2077	1	C33H34N5O9S	676.2083	0.9	0.6	38.1	0.2

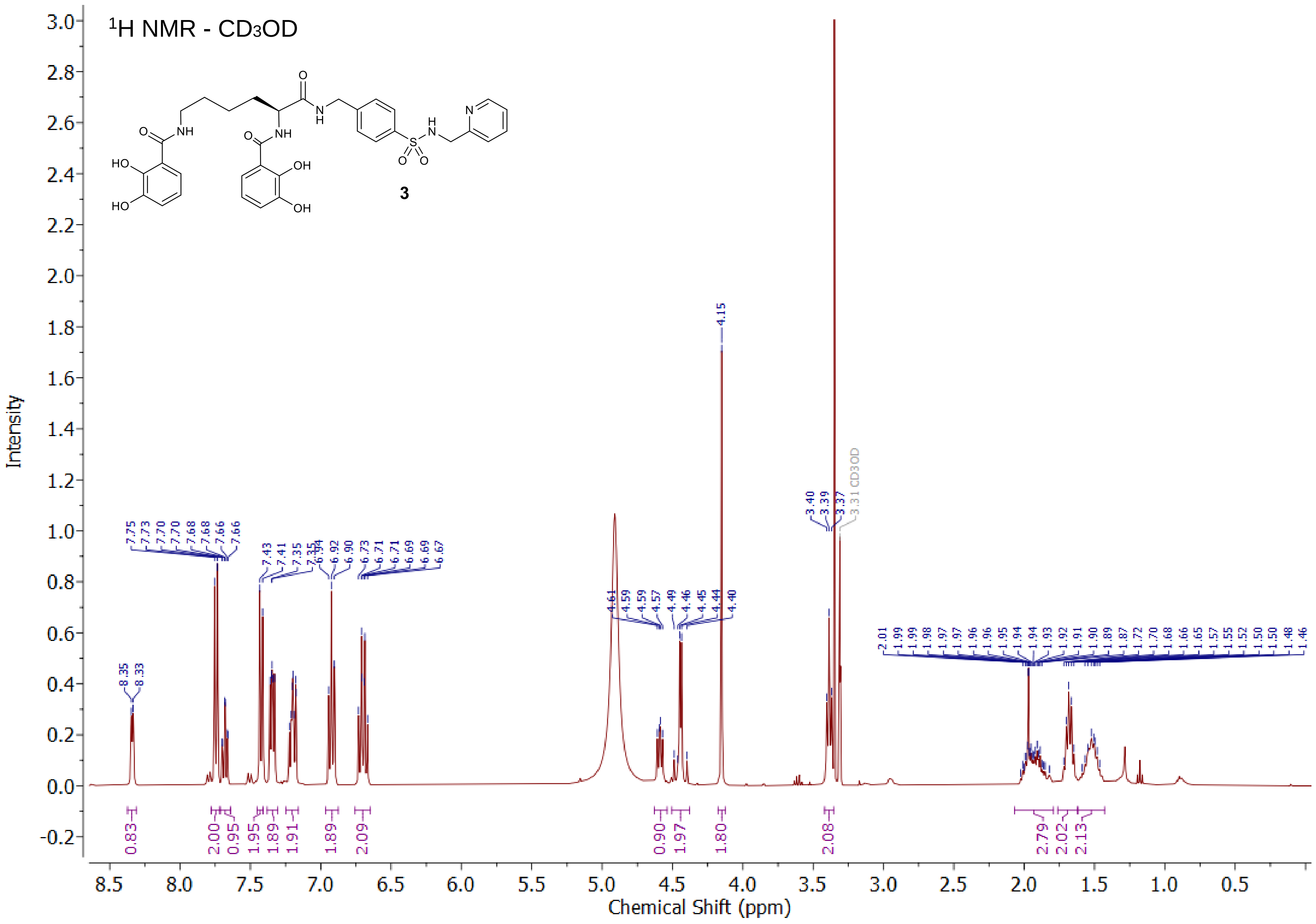
1H NMR - CD3OD
3
Intensity
Chemical Shift (ppm)

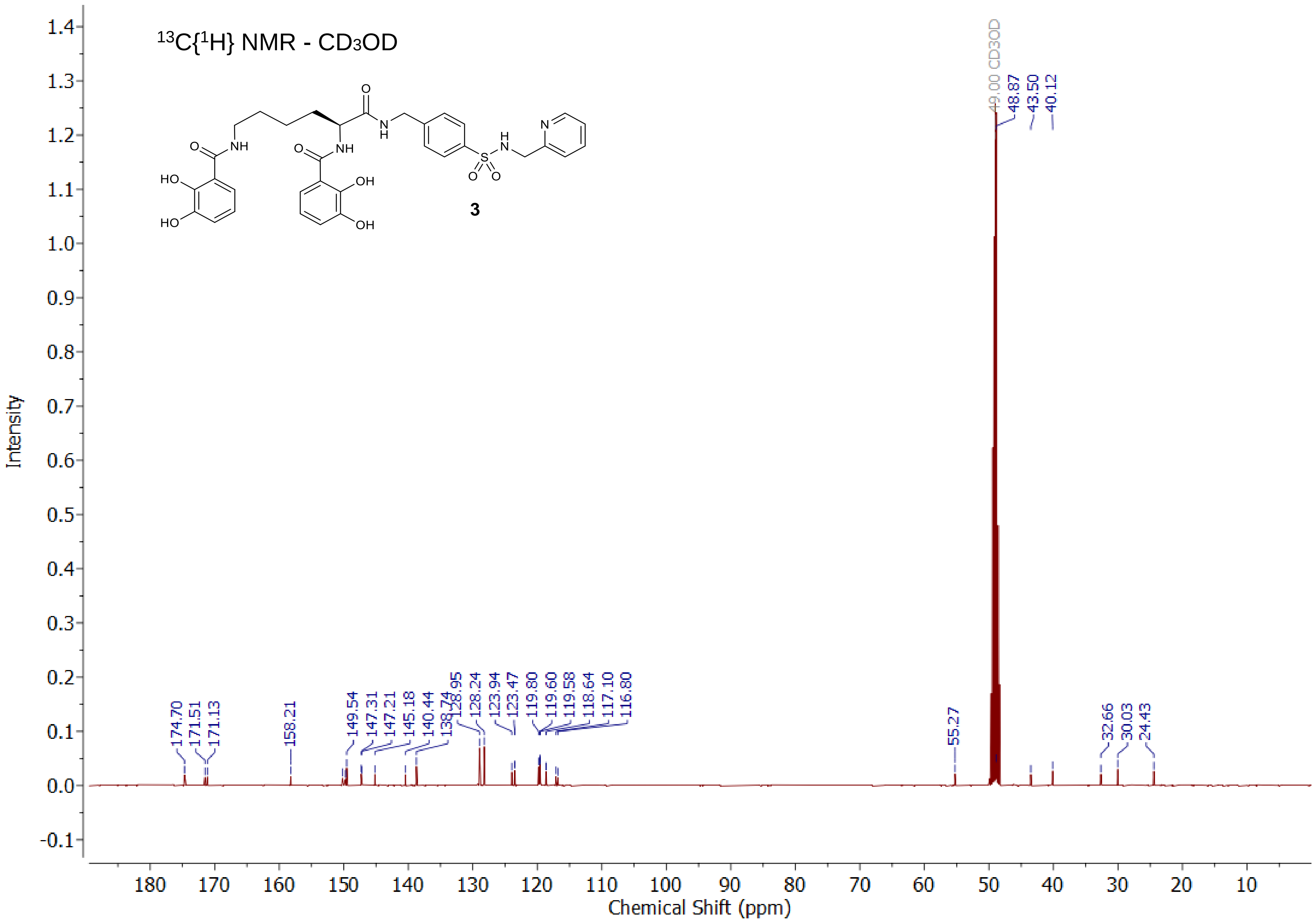

13C{1H} NMR - CD3OD
3
174.70
171.51
171.13
158.21
149.54
147.31
147.21
145.18
140.44
138.74
128.95
128.24
123.94
123.47
119.80
119.60
119.58
118.64
117.10
116.80
55.27
49.00 CD3OD
48.87
43.50
40.12
32.66
30.03
24.43
Intensity
Chemical Shift (ppm)

11. Supplementary references

(1) White, C.; Yates, A.; Maitlis, P.; Heinekey, D. (η5-Pentamethylcyclopentadienyl) Rhodium and-Iridium Compounds. *Inorganic Syntheses* **1992**, 228-234.

(2) Raines, D. J.; Clarke, J. E.; Blagova, E. V.; Dodson, E. J.; Wilson, K. S.; Duhme-Klair, A.-K. Redox-switchable siderophore anchor enables reversible artificial metalloenzyme assembly. *Nature Catalysis* **2018**, *1* (9), 680-688.

(3) Blagova, E. V.; Miller, A. H.; Bennett, M.; Booth, R. L.; Dodson, E. J.; Duhme-Klair, A.-K.; Wilson, K. S. Thermostable homologues of the periplasmic siderophore-binding protein CeuE from Geobacillus stearothermophilus and Parageobacillus thermoglucosidasius. *Acta Crystallographica Section D* **2023**, *79* (8), 694-705.

(4) Stein, A.; Chen, D.; Igareta, N. V.; Cotelle, Y.; Rebelein, J. G.; Ward, T. R. A Dual Anchoring Strategy for the Directed Evolution of Improved Artificial Transfer Hydrogenases Based on Carbonic Anhydrase. *ACS central science* **2021**, *7* (11), 1874-1884.

(5) Fujii, A.; Hashiguchi, S.; Uematsu, N.; Ikariya, T.; Noyori, R. Ruthenium (II)-catalyzed asymmetric transfer hydrogenation of ketones using a formic acid− triethylamine mixture. *Journal of the American Chemical Society* **1996**, *118* (10), 2521-2522.
